# Towards synthetic biological approaches to resource utilization on space missions

**DOI:** 10.1098/rsif.2014.0715

**Published:** 2015-01-06

**Authors:** Amor A. Menezes, John Cumbers, John A. Hogan, Adam P. Arkin

**Affiliations:** 1 California Institute for Quantitative Biosciences, University of California, 2151 Berkeley Way, Berkeley, CA 94704-5230, USA; 2 NASA Ames Space Portal, NASA Ames Research Center, MS 555-2, Moffett Field, CA 94035, USA; 3 Bioengineering Branch, NASA Ames Research Center, MS 239-15, Moffett Field, CA 94035, USA; 4 E.O. Lawrence Berkeley National Laboratory, 1 Cyclotron Road, MS 955-512L, Berkeley, CA 94720, USA; 5 Department of Bioengineering, University of California, Berkeley, CA 94720, USA

**Keywords:** space synthetic biology, methanogens, cyanobacteria, *Spirulina*, polyhydroxybutyrate, acetaminophen

## Abstract

This paper demonstrates the significant utility of deploying non-traditional biological techniques to harness available volatiles and waste resources on manned missions to explore the Moon and Mars. Compared with anticipated non-biological approaches, it is determined that for 916 day Martian missions: 205 days of high-quality methane and oxygen Mars bioproduction with *Methanobacterium thermoautotrophicum* can reduce the mass of a Martian fuel-manufacture plant by 56%; 496 days of biomass generation with *Arthrospira platensis* and *Arthrospira maxima* on Mars can decrease the shipped wet-food mixed-menu mass for a Mars stay and a one-way voyage by 38%; 202 days of Mars polyhydroxybutyrate synthesis with *Cupriavidus necator* can lower the shipped mass to three-dimensional print a 120 m^3^ six-person habitat by 85% and a few days of acetaminophen production with engineered *Synechocystis* sp. PCC 6803 can completely replenish expired or irradiated stocks of the pharmaceutical, thereby providing independence from unmanned resupply spacecraft that take up to 210 days to arrive. Analogous outcomes are included for lunar missions. Because of the benign assumptions involved, the results provide a glimpse of the intriguing potential of ‘space synthetic biology’, and help focus related efforts for immediate, near-term impact.

## 1. Introduction

Manned space exploration missions deploy technologies and products that mitigate crew-safety concerns and that assist with mission accomplishment. These technologies are continuously evaluated for relevance and cost, a term that accounts for launch mass, drawn power, volumetric size, useful product life, astronaut utility, etc. This evaluation is important, because space missions are inherently expensive; every unit mass of payload that is launched into space necessitates the launch of an additional 99 units of mass [1]. Hence, there is an interest in novel technologies that simultaneously decrease cost, reduce risk (through redundancy in process or output) and increase the probability of mission success. Promising new technologies are presently sought by the National Aeronautics and Space Administration (NASA) for

— generating fuels to propel primary spaceship and supplementary personal conveyances [2–6];
— manufacturing components in space (e.g. by three-dimensional printing [7–11]);
— replacing cargoes of pre-packaged food (for instance, printing food [12,13]);
— developing redundant life support systems for gas purification, water treatment, etc. [14–16];
— tackling diseases and increasing the shelf-life of therapeutics and pharmaceuticals [17–21];
— sensing and monitoring radiation levels and shield strength with light-weight apparatus [22,23]; and
— recycling trash (e.g. as three-dimensional printing media [8] or to augment radiation shields after heat melt compaction [24]).

Typically, the cost of these new technologies is reduced through *in situ* resource utilization (ISRU) [25] which consists of harnessing materials located at a mission's destination.

This paper investigates how current biological techniques and future synthetic biology [26] progress can meet several of the above-mentioned needs. The work reviews existing biological processes to demonstrate that they already constitute a competitive yet non-traditional technology that is capable of processing volatiles and waste resources readily available on two representative space missions in a way that reduces the launch mass of propellant, food and raw material for three-dimensional printing, and also overcomes the decreased product shelf-life of a common therapeutic. The paper employs these reviewed processes in designs for natural and artificially enhanced biological manufacturing strategies that can be leveraged to satisfy space input-availability and output-desirability constraints. The work then analyses methodological feasibility, technique versatility and the costs and yields of feedstocks and constituents, and compares possible future ‘space synthetic biology’ advances to other new aerospace technologies.

Although a novel technique, synthetic biology has already been tapped for its potential to eliminate plastic waste [27], enrich food [28,29], monitor pollution and chemicals [30] and be an ISRU tool [31]. This paper furthers these forays and widens the scope of the technology by indicating its capacity for extensive product applicability in space despite the severe input limitations imposed by the space environment.

Accordingly, this paper will

(1) identify desirable endpoints on future space missions that can be tackled with biological techniques;
(2) review the expected cost of producing these endpoints with novel, non-biological technologies, where cost is defined in terms of either launch mass or usable life; and
(3) propose feasible biological approaches to producing these endpoints, compute the yield of these approaches, quantify the resultant cost benefits that are afforded by these approaches and discuss improvements that are suggested by synthetic biology. In parallel, this objective will help ascertain high-impact synthetic biology molecules and processes for space exploration.

The inspiration for this study is the US Department of Energy (DOE) report on the top value-added chemicals from biomass [32], where similar objectives were considered for a less restrictive version of the problem with different production inputs (sugars) and endpoints (biorefinery usage).

The remainder of this work is as follows. Section 2 details the type of mapping from resource inputs to mission product outputs that is sought by this work. Sections 3–6 contrast a biological version of this mapping with a satisfactory non-biological approach that is currently under consideration by the space community. Section 7 summarizes the organisms, processes and products that are deemed important for space synthetic biology. Section 8 presents concluding remarks.

## 2. Problem definition and solution approach

### 2.1. Input availability on sample space missions

**Figure 1.**
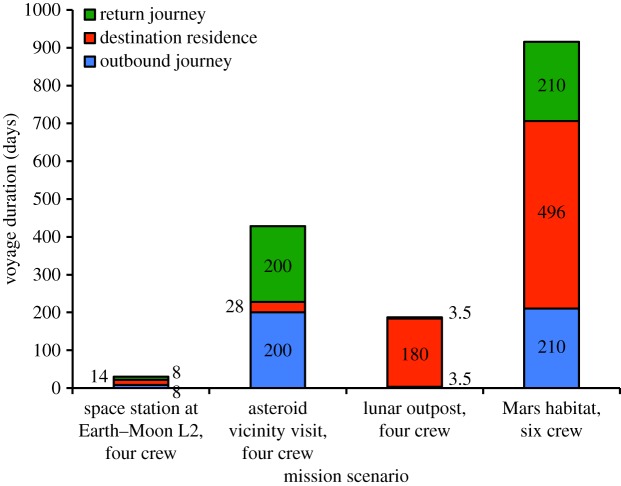
Projected Moon and Mars missions have the greatest destination residence times, thereby necessitating *in situ* resource utilization when at the destination on these missions to reduce costs and risk. The Mars mission also has the longest journey times, comparable to that of the asteroid visit mission; thus, effective resource utilization during Mars mission travel is also necessary, with any analysis applicable also to the asteroid mission.

Recent space policy roadmaps [33–35] suggest four candidate, near-future, manned space ventures (figure 1), namely

(1) a four-person, 30 day return voyage to a space station positioned at the Earth–Moon L2 Lagrange point, comprising 8 days of travel each way and a 14 day station stay [36,37] (effectively yielding 30 days of voyage-generated resources, i.e. crew-produced waste, and 0 days of non-travel-related resources);
(2) a four-person, 428 day return voyage to an asteroid, averaging 200 days of travel each way and a 28 day stay in the asteroid vicinity (this is the full-capability, high-energy mission in [38], which effectively yields 428 days of voyage-generated resources supplemented by 28 days of asteroid-related resources);
(3) a four-person, 187 day return voyage to the Moon, comprising 3.5 days of travel each way and a 180 day stay at a previously established lunar outpost [39,40]; and
(4) a six-person, 916 day return voyage to Mars, comprising 210 days of travel each way and a 496 day stay on a Martian surface habitat (the conjunction class long-stay mission in [41]).

Of these candidate missions, the fourth warrants an analysis of applicable bioproduction techniques for two reasons: a lengthy total time spent on board a spacecraft that is comparable to the long residence time of the asteroid-investigation mission, and an extremely lengthy stay on Mars that makes it vital to explore all technologies that could reduce risk, decrease launch mass and manufacture products with a short shelf-life. Given its possible precursor status for the Martian mission, the lunar mission will also be examined for biomanufacturing benefits.

Representative values of the masses of crew-produced wastes, which serve as potential resources for biology-based designs, are listed in table 1 [42,43] for the previously stated Martian- and lunar-manned space exploration missions. Further inputs for these two missions can be drawn from the Mars atmosphere or the permanently shadowed craters on the south pole of the Moon, respectively (table 2; [31,44,45]), and the Martian soil or lunar regolith, respectively (table 3; [31,46]).

**Table 1.** Masses of crew-generated wastes that could be resources for synthetic biology applications.

waste	unit mass produced [42] (kg per crew per day)	mass produced, six outbound to Mars (kg)	mass produced, six living on Mars (kg)	mass produced, four outbound to Moon (kg)	mass produced, four living on Moon (kg)
carbon dioxide ⇒ carbon content ⇒ oxygen content	1 0.273 0.727	1260 344 916	2976 812 2164	14 3.82 10.18	720 196 524
urine water ⇒ nitrogen content (ammoniacal) [43]	1.5 0.012	1890 15	4464 36	21 0.17	1080 9
non-recycled water (assuming recapture) ⇒ hydrogen content ⇒ oxygen content	2.37 0.266 2.104	2986 335 2651	7053 791 6262	33.18 3.72 29.46	1706 191 1515

**Table 2:** Martian atmospheric gases and lunar volatiles that could be resources for synthetic biology applications.

compound	Mars atmospheric content [44] (%)	Moon pole shadowed crater ejecta plume content [31,45] (wt%)
carbon dioxide	95.32	0.12
nitrogen	2.7	
argon	1.6	
oxygen	0.13	
carbon monoxide	0.07	0.023
water vapour	0.03	5.6
nitric oxide	0.013	
hydrogen sulfide		0.94
ammonia		0.34
sulfur dioxide		0.18
ethylene		0.17
methanol		0.09
hydrogen		0.047
methane		0.04
hydroxide		0.002

**Table 3.** Martian soil and lunar regolith compounds that could be resources for synthetic biology applications.

compound	mean Mars soil composition per Mars exploration rover *Spirit* [46] (wt%)	mean lunar regolith composition [31] (wt%)
silicon dioxide (SiO_2_)	45.8	47.3
ferric oxide (Fe_2_O_3_)	17.6	
alumina (Al_2_O_3_)	10.0	17.8
magnesium oxide (MgO)	9.3	9.6
calcium oxide (CaO)	6.1	11.4
sodium oxide (Na_2_O)	3.3	0.7
titanium dioxide (TiO_2_)	0.81	1.6
potassium oxide (K_2_O)	0.41	0.6
ferrous oxide (FeO)		10.5
chromic oxide (Cr_2_O_3)_		0.2
manganous oxide (MnO)		0.1

The tables suggest that carbon dioxide (CO_2_) and nitrogen (N_2_) are somewhat plentiful resources for biology applications over the course of a Mars voyage and stay. However, these resources are significantly scarcer on a Moon mission. Yet, if large enough excavators and bioreactors are deployed (see [31] and §3.2.1), there should be enough of these resources extracted to test the viability of biological techniques prior to a Mars voyage. Hydrogen (H_2_) and oxygen (O_2_) may also be available for biomanufacturing on both missions as a result of the electrolysis of polar water, but it is expected that water availability will be reduced given its priority to support crew life. If required, hydrogen can be transported to Mars and also stored until use, but this process is considered somewhat difficult and problematic [44]. On the Moon however, hydrogen is already present [47]. Oxygen may also be harvested from the Martian soil or the lunar regolith with post-excavation processing. Hence, bioproduction applications for Mars and the Moon need to take as inputs: carbon dioxide, nitrogen (either nitrogen gas, ammoniacal nitrogen, ammonia or nitric oxide), hydrogen and oxygen, ordered here by their availability. This resource set of elements and simple compounds can conceivably support biological systems because its constituent elements form a subset of the main elements required for life, namely carbon, hydrogen, nitrogen, oxygen, phosphorus and sulfur. The latter two elements are not readily available on both Martian and lunar missions, although sulfur is present on the Moon alone (table 2). Soil-based metal resources from Mars and regolith-derived metal resources from the Moon are not considered suitable for biology application in this paper.

### 2.2. Desirable outputs for sample space missions

Prospective biology targets are those that are most mission-relevant and expensive. A cost-based ordering of the items required for a Mars or Moon mission cannot be compiled, because cargo manifests for these future missions are still in flux. Moreover, at this early stage of mission planning, the form of the cost metric itself and the relative weightings it contains is somewhat unclear. This is attributable to changing technology-readiness-levels (which correspondingly change weightings that are based on manufacturing novelty and production-associated uncertainty in an evaluated property like launch mass) and disparate evaluation criteria (e.g. shelf-life versus mass: a crew health article that weighs little at launch, but expires quickly is arguably as important on a long-duration mission as an item that has significant launch mass).

Nevertheless, four targets quantifiably stand out for biological production. Fuel, for instance, is currently projected to be ‘about two-thirds of the mass on an Earth-to-Mars-to-Earth mission [, and] cost-effective [extraterrestrial]-produced propellant could decrease the mass that must be lifted from Earth by a factor of two to three’ [33]. Food is another necessary target, as evidenced by crew meals constituting the bulk (306 kg/520 kg or 59%) of a recent supply mission to the International Space Station (ISS) [48].

Biopolymers are a third target, because plastics are included in the list of feedstock materials that can be used for three-dimensional printing [49]. The three-dimensional printing of structures to manufacture a spacecraft in space can decrease roughly 30% of the craft's launch mass by reducing the supporting structural material that is required [49], and additive manufacturing can also reduce the launch mass cost associated with storing a multitude of spacecraft spare parts. Because the 30% number presumes a launch of necessary printer media, the extraterrestrial production of raw material for three-dimensional printing, e.g. biopolymers, can achieve even greater mass reductions. Further savings can also be realized by deploying additive manufacturing for other purposes, such as the construction of habitats [10,11], rocket engine parts [9], sample containers [8], spacecraft electronic platforms [7], etc.

Lastly, the accelerated expiration of pharmaceuticals induced by space radiation [19–21] (less than 50% acceptability of solid dosage forms after 596 days in space and 27% acceptability of solid formulations after 880 days in space [19]) necessitates the on-demand synthetic manufacture of such pharmaceuticals on long-duration missions. In this paper, a versatile drug to treat infection and pain symptoms, e.g. aspirin, acetaminophen, etc., is targeted for biosynthesis. It is envisioned that this drug will be manufactured when desired by astronauts using bacteria that are activated from a frozen state. The bacteria will not themselves ‘expire’ from space radiation because of storage in a small, lead-lined container while inactive; bacterial spores and rock-colonizing eukaryotes can survive with little protection in space [50,51] for between 1.5 and 6 years [52–54]. Quality control of astronaut-activated bacteria can be performed through portable gene sequencers that are in development [55], and that are already being contemplated for use in space exploration [56].

The choice of four targets outlined in this section is further justified by their inclusion in the list of needs presented in §1 for which NASA seeks promising new technologies. Sections 3–6 confirm the feasibility and benefits of producing each of these desirable endpoints with contemporary biological techniques.

### 2.3. Problem specification

**Figure 2.**
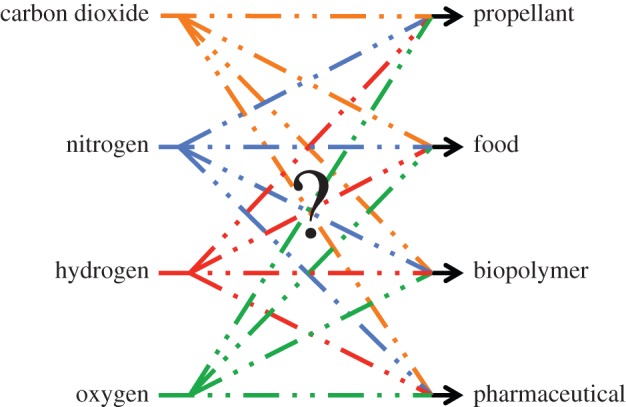
The biology mapping that is sought in this paper is from a constrained set of inputs (carbon dioxide, nitrogen, hydrogen and oxygen) to a fixed set of output classes (propellant, food, biopolymer, pharmaceutical) where the exact output in each class (e.g. propellant chemical name) is unspecified. The map is unconstrained in the type or number of intermediates that is required to manufacture one or more outputs, but it is desired that the number of these intermediates be minimized and that the use of any intermediate be kept in common as far as possible to ensure that the production of all outputs is efficient.

Hence, the design problem that is tackled in this paper: design biology processes to go from the inputs listed in the left column of figure 2 to the outputs listed in the right column of figure 2, using the fewest number of intermediates, organisms and steps, with the greatest possible commonality of such intermediates, organisms and steps, and with the goal of substantially reducing launch mass and increasing product shelf-life.

The availability of only a few input elements and simple compounds coupled with the predefined desirability of various output products constrain space biology designs. The current state of the technology requires design options to include, as a first step, those organisms that already use the same resources on the Earth. Thereafter, the outputs of these organisms can serve as inputs to other organisms. The yields of such modular designs can be analysed and then improved upon with bioengineering and genetic modification techniques. As synthetic biology matures over the coming decades, it may be possible to build designer organisms from scratch that directly manufacture the desired products efficiently.

### 2.4. Problem solution philosophy

Because carbon dioxide and nitrogen compounds are the dominant available resources, organisms that harness these resources and the yields and efficiencies at which they do so are of prime importance. Further, the outputs of these organisms will be useful as either the desirable targets of figure 2 or as feedstock intermediates to obtain these targets. Thus, we summarize in electronic supplementary material, table S1 the mechanisms of action and the outputs produced by organisms that take in carbon dioxide, as detailed by [57]. Electronic supplementary material, table S2 provides a similar summary of organisms that use and produce various nitrogen compounds; these organisms also play a role in the microbial nitrogen cycle [58–62].

A greedy design approach involves employing the lowest-energy carbon dioxide fixation process from electronic supplementary material, table S1, which exists in methanogens and acetogens. Conveniently, the responsible pathway in these organisms, the Wood–Ljungdahl pathway, requires the input of hydrogen and the presence of anoxic conditions. We temporarily defer discussion of methanogens and methane production to §3.2.1. Acetate, produced by acetogens, however, has the potential to be an extremely useful feedstock, as it is both a fundamental component of the (aerobic metabolism) citric acid cycle and a precursor for biosynthetic targets such as biofuels as described in §2.5.

**Figure 3.**
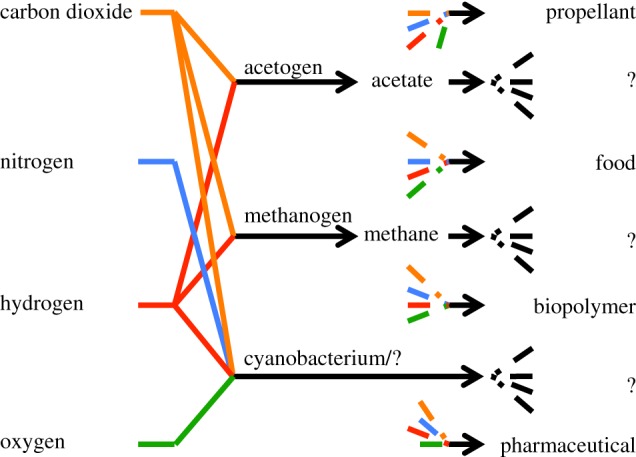
This paper limits the sought biological map of figure 2 to single-steps (from one or more of the four input resources on the left side of the figure, e.g. by deploying cyanobacteria) or single-intermediate (e.g. acetate) processes, so that a greedy approach to produce the desired intermediates and outputs is optimally efficient. Although not illustrated, the biological transformation of the intermediates into outputs can also make use of available resources.

In what follows, acetate is taken to be the single available feedstock for all modular bioproduction designs, selected for its high CO_2_-fixation efficiency and biomanufacture viability (see §2.5). Our design philosophy involves (figure 3)

(1) using acetogens and methanogens to efficiently convert carbon dioxide directly into a desirable output; or
(2) using organisms that metabolize efficiently produced acetate to efficiently convert nitrogen or nitrogen compounds directly into a desirable output; or
(3) harnessing other organisms to convert carbon dioxide directly into a desirable output when deemed advantageous.

An example phylum for the latter that is explored in the recent reviews [63–67] is cyanobacteria. Advantages of deploying cyanobacteria include well-developed genetic tools; sequenced, annotated and small genomes of model organisms; relatively rapid growth and the capacity to use nitrogen gas directly [65]. This last advantage is also apparent from cyanobacteria's appearance in electronic supplementary material, tables S1 and S2.

Once biomanufacture strategies are formulated according to the abovementioned design philosophy, we examine volumetric yields and the cost-effectiveness of each suggested process. Bottlenecks that decrease yield or increase cost are identified, and we propose synthetic biology approaches to improve strategy performance.

### 2.5. Rationale for problem solution philosophy

It has been known for more than 25 years that acetate can be produced from hydrogen and carbon dioxide by the microflora of human faeces [68], and is therefore a useful intermediate for closed life-support systems in space. Recently, acetogens have garnered attention for their synthetic biology and biofuel potential [69,70], with [70] demonstrating that the Wood–Ljungdahl pathway is the most efficient non-photosynthetic CO_2_ fixation pathway to produce acetate with a hydrogen substrate: approximately 95% (as summarized by Lovley & Nevin [71]) of the carbon and electron flow is diverted to the production of small organic, cell-excreted products. The theoretical yields for maximum acetate production were stoichiometrically calculated in [70] to be 23.6 mol per 100 mol H_2_ (using 49.6 mol of CO_2_) and 47.5 mol per 100 mol CO_2_ (using 201.5 mol of H_2_). The current experimental maximum volumetric productivity through autotrophic growth is 7.4 g of acetate per litre per day at a partial hydrogen pressure of 1700 mbar with *Acetobacterium woodii*, and the final acetate concentration of 44 g l^−1^ measured after 11 days at a constant process pH of 7.0 represents the highest reported value up to 2010 [72]. Using the reported gas flow rate of 15 l h^−1^, Henry's law constant for the dissolved concentration of hydrogen of 0.749 mmol l bar^−1^, and a 1 l working volume in a 2 l tank [72], the experimental rate of 7.4 g of acetate per litre per day corresponds to acetate production of 27.3 mol per 100 mol H_2_, which is only slightly higher than the theoretical maximum. Hence, it is possible to efficiently fix large quantities of carbon dioxide into acetate with one or more continuous stirred tank bioreactors.

An alternative acetate bioproduction technique [71,73–77] is one in which electrical energy is used to provide microbes with low-potential electrons that facilitate carbon dioxide reduction and cellular processes. With this technique, either microbial biofilms grow on electrodes and directly accept electrons from them, or a traditional electron donor such as H_2_ is electrochemically generated from, for instance, water. This technique has yet to achieve the levels of autotrophic acetate production—the latest reports are of 1.04 g of acetate per litre per day and 10.5 g l^−1^ over 20 days [77]—but the method seems capable of coulombic (electron) efficiencies up to 97% when producing a mixture of methane and acetic acid [75].

## 3. Propellant generation in space

### 3.1. Current propellant generation method and cost

Common liquid chemical rocket propellants include hydrazine (N_2_H_4_), which can be employed as both a monopropellant and as a bipropellant when mixed with an oxidizer; oxidizers liquid oxygen (O_2_, abbreviated LOX), hydrogen peroxide (H_2_O_2_), nitrogen tetroxide (N_2_O_4_, abbreviated NTO) and mixed oxides of nitrogen (a mixture of NTO with nitric oxide, abbreviated MON); hydrocarbon fuels such as methane (CH_4_) and kerosene-based RP-1; liquid hydrogen (H_2_, denoted LH_2_) and bipropellant organic compounds of hydrazine such as monomethylhydrazine (CH_3_NHNH_2_, abbreviated MMH) and unsymmetrical dimethylhydrazine ((CH_3_) _2_NNH_2_, abbreviated UDMH) [1,78–82]. Hydrogen, while a fuel capable of imparting a very high specific impulse (a measure of provided thrust), is the least dense of all known fuels, and therefore requires specialized combustion apparatus [79,82]. Trade studies over the past 15 years have identified ‘Mars atmospheric CO_2_ [as] important for O_2_ and hydrocarbon fuel production, nitrogen (N_2_) [as] important as a buffer gas for life support, [and] argon (Ar) [as] useful as an inert gas for science experiments and propulsion system purging’ [83]. Accordingly, carbon-based fuels such as methane, methanol, benzene/toluene and Fischer–Tropsch products have been closely examined for their Mars utility [84]. Of these, a (liquefied) methane–(liquefied) oxygen combination is the currently selected ascent propellant, based upon its propulsion performance and *in situ* production process complexity [84,85]. Methane's density-specific impulse is higher than that of hydrogen, which is an important consideration for compact, low-volume engine feasibility. Yet, methane is not so dense that it is correspondingly too costly (in terms of mass) for a Mars ascent. A comprehensive comparison of the properties of various propellants, such as specific gravity, specific impulse, etc., is provided in [1,79].

About 30–40 T of methane–oxygen propellant is required to lift a Mars ascent vehicle to orbit [44], and there are three ways to produce the requisite amount under different assumptions as described in [84]. The first option is to deliver methane fuel directly from Earth and produce oxygen alone on Mars; the estimated mass cost for this option is 7512 kg. A second estimate for the production of both methane and oxygen on Mars assumes the delivery of hydrogen, and the total mass cost for this scenario is 3251 kg. An alternative option makes use of water already present in the Martian soil at either 3 wt% (i.e. 3% by weight) or 8 wt%, and the shipped mass costs for these conditions are 2658 and 2021 kg, respectively. Electronic supplementary material, table S3 details the production mechanisms and mass requirements for each of these scenarios.

Ten tonnes of a similar fuel mix are required for a lunar module ascent [44], and the associated mass costs for two different production options are also listed in electronic supplementary material, table S3. These options are similar to those for Mars in that the first stipulates the delivery of hydrogen, whereas the second uses available water harvested from lunar regolith. The estimated mass cost of the first scenario depends upon the availability of ferrous oxide in the lunar regolith, and is 4060 kg if this availability is 5 wt% and 3681 kg if this availability is 14 wt%. For the second scenario of water-harvesting, the shipped mass cost is 1807 kg. Both these lunar scenarios require a cargo of carbon, but this carbon can be recycled from packaging materials and other trash.

There exists another rocket fuel candidate with specific impulse and density-specific impulse properties that are comparable to a methane–oxygen combination: the currently uncommon blended monopropellant, nitrous oxide–hydrocarbon, where the hydrocarbon is propane, ethane, ethylene, acetylene, etc. The generation cost for this alternative propellant is determined in the electronic supplementary material, §S2.

### 3.2. Biological propellant generation methods and costs

Biological production of common liquid chemical rocket propellants on and *en route* to Mars is conceivable based on the compatibility of their elemental composition with available inputs (see §2.1), but compounds that are toxic cannot be synthesized by organisms, and compounds that are complex require correspondingly involved biomanufacturing processes. Consequently, RP-1, MMH, UDMH, hydrazine, NTO and MON may be eliminated from bioproduction consideration. The biomanufacture of hydrogen peroxide can also be ruled out, because the relatively efficient bioelectrochemical process with wastewater bacteria that produces this compound [86] requires both oxygen, which has the lowest ready-availability as explained in §2.1, and acetate, which requires transported hydrogen; hence, it would be more logical to transport hydrogen peroxide directly if deemed necessary. Thus, the only feasible candidate for biosynthesis among the commonly used liquid propellants is methane. The biomanufacture of both methane–oxygen and nitrous oxide–hydrocarbon is examined next. We make use of the approximate empty-mass costs for various bioreactor sizes [87] presented in table 4.

**Table 4.** Sample mass, power and volume costs of off-the-shelf bioreactors.

max. working volume (l) [87]	min. working volume (l) [87]	empty mass (kg) [87]	mean power input/working volume at 0.02 vvm aeration and constant shear (mW l^−1^) [88]	skid volume (m^3^) [87]
50	12	165	7.665	0.710
200	40	239	5.978	1.553
500	100	333	4.082	2.202
1000	200	446	3.240	3.356
2000	400	794	2.407	4.948

#### 3.2.1. Methane–oxygen

**Figure 4.**
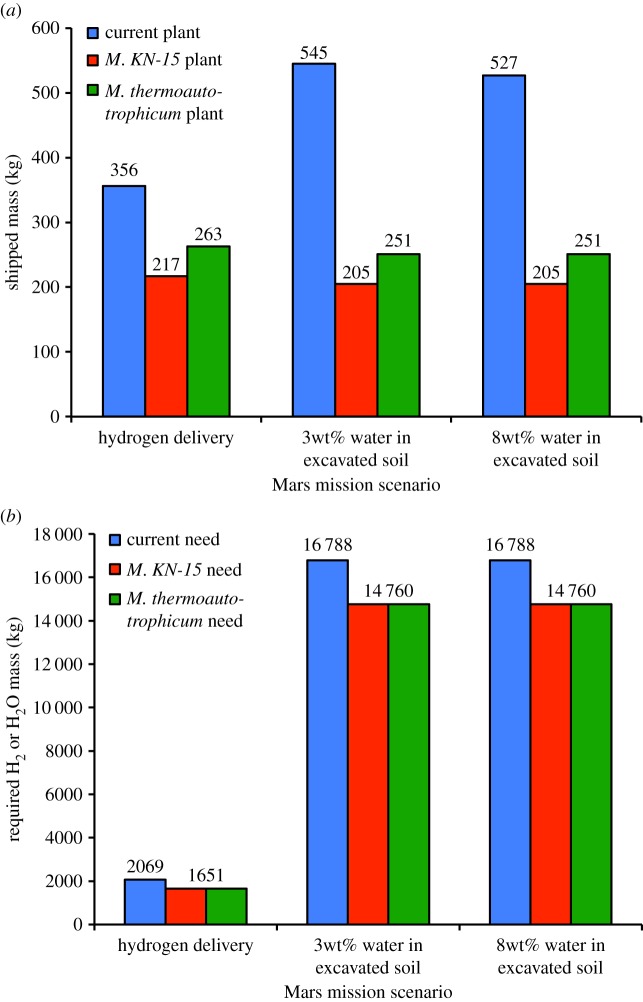
The mass of the Mars atmosphere processing plant and required hydrogen or water input in each of the three Design Reference Architecture 5.0 mission scenarios [84] can be reduced by using a *Methanobacteria* (either *KN-15* or *thermoautotrophicum*) bioreactor instead of a Sabatier reactor. (*a*) Atmosphere processing plant and (*b*) required hydrogen or water input.

Similar to acetate biosynthesis, there are electrochemical (see [89] and the references in [71]) and bioreactor (see the recent review [90] and references therein) approaches to producing methane from carbon dioxide and hydrogen with methanogens. According to [90], Nishimura *et al*. [91] achieves the highest continuous stirred tank reactor volumetric productivity with the thermophile strain *KN-15* of *Methanobacterium* at 1280 mmol l^−1^ h^−1^, which corresponds to a production rate of 492.8 g of methane per litre per day. However, the methane offgas concentration with this organism is 18.3% [90], necessitating considerable downstream treatment to increase gas purity. Reference [92] reported a high of 96% methane offgas concentration in a continuous stirred tank reactor with *Methanobacterium thermoautotrophicum*, strain Marburg, producing methane at 530 mmol l^−1^ h^−1^ [90], which corresponds to a production rate of 204.1 g of methane per litre per day. Electronic supplementary material, table S4 summarizes the Mars ISRU mass cost of these organisms when a flow rate constraint of 3000 g of water h^−1^ is imposed by incorporating a 6 kg electrolyser that was recently validated in [93]. Significant reductions in the mass of methane–oxygen production-plants are indicated. With *M. thermoautotrophicum*, the plant mass can be reduced by 26% if hydrogen delivery to Mars is assumed (and the reverse water gas shift (RWGS) process is included along with another electrolyser, so as to together increase the oxygen manufacture rate), whereas a 56% and a 55% mass reduction is possible for the cases where water is present in the Martian soil at 3 and 8wt%, respectively (figure 4). The estimated completion time for the propellant generation process is 205 days, about 41% of Mars residence time. Because the bioreactor can be projected to draw 938.5 mW of power [88] and occupy a volume of 1.553 m^3^ (table 4), the power reduction is nearly 100% for all scenarios, but the volume is increased fivefold in the hydrogen delivery case, 1.5-fold in the 3 wt%-water case, and more than twofold in the 8 wt%-water case.

**Figure 5.**
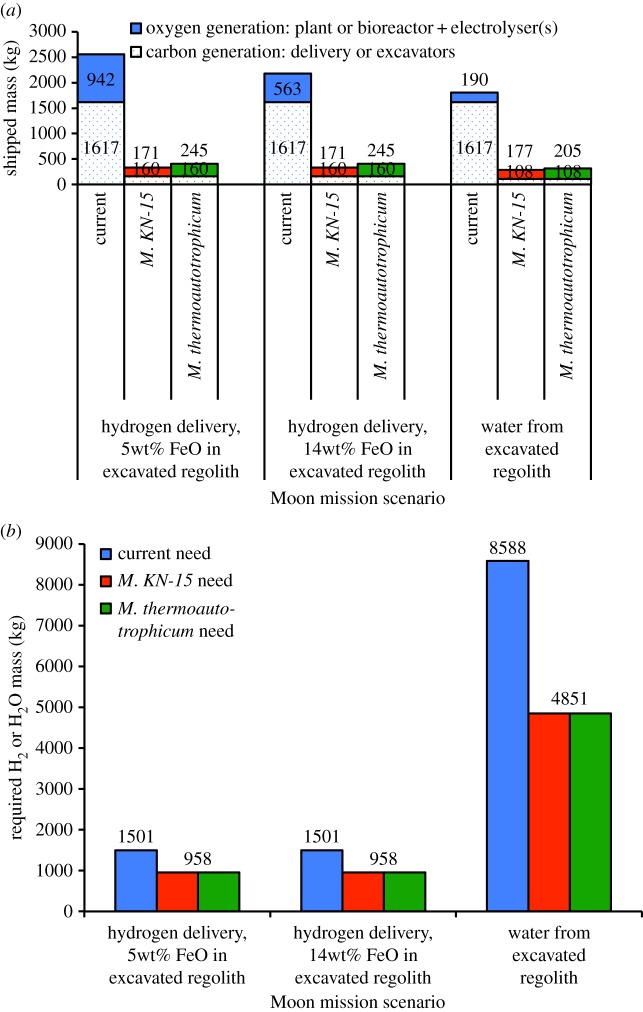
The mass of the Moon oxygen and carbon generation mechanisms and required hydrogen or water input in each of the lunar mission scenarios can be reduced by using a *Methanobacteria* (either *KN-15* or *thermoautotrophicum*) bioreactor. (*a*) Oxygen and carbon generation mechanisms and (*b*) required hydrogen or water input.

Limited carbon dioxide availability on the Moon (0.12% in the permanently shadowed craters of the south pole as indicated in table 3) can bias aerospace engineering designs away from excavations of large quantities of polar regolith for carbon dioxide processing towards the utilization of cargoes of carbon (i.e. packaging materials and trash). However, the former option is achievable in practice, because an 80 kg excavator in the 2009 NASA Lunar Regolith Challenge lifted 500 kg of simulated regolith into a container in 30 min [94]. Assuming complete carbon dioxide extraction from the excavated regolith, it follows that at least 28.8 kg of carbon dioxide is available per day per excavator at current excavation rates. Stoichiometry then implies the possibility of a daily production of 10.5 kg of methane per excavator. For a scaled-up 108 kg excavator (electronic supplementary material, table S5), using *M. thermoautotrophicum* to produce over 14 kg of methane per day, a 70 l working volume is required. This volume can be contained in a 100 l bioreactor, which has an estimated empty mass cost of 193 kg. Together with two 6 kg electrolysis units, one for processing bioreactor output and another for processing excavated regolith, up to 83% in mass reduction is possible compared with current carbon delivery and oxygen generation methods (figure 5). According to table 4, the estimated bioreactor drawn power is 497.2 mW, and the estimated volumetric size is 0.991 m^3^. The methane production process can be calculated to take 153 days to complete.

The 6 kg of carbon dioxide that is produced daily by crew members during a Martian mission can also be converted into methane to power supplementary personal conveyances like jetpacks for emergency use, or to reduce the time required for methane production on Mars. The stoichiometry suggests that about 2.19 kg of methane can be produced per day, and the use of *M. thermoautotrophicum* for generating methane requires a working volume of less than 11 l in the bioreactor. The mass cost of this emergency option is then 165 kg, as suggested by a 50 l bioreactor (171 kg with an electrolyser unit). Per [88] and table 4, this bioreactor draws 84.32 mW of power and requires 0.710 m^3^ of space. The requisite hydrogen for this bioreactor is supplied by electrolysed water that is output by the reaction, supplemented by hydrogen from onboard stores. These stores can be replenished by the electrolysis of water on Mars. Using data from table 1, 459 kg of methane can be generated over the course of a one-way 210-day journey, and 1085 kg of methane can be generated from the total carbon dioxide produced during Martian residence.

Electronic supplementary material, table S6 presents the unoptimized mass cost of nutrients and growth media, when extrapolated from the literature requirements, for Mars-based methane bioproduction over the anticipated manufacture period of 205 days. These nutrients and growth media are overly rich, and require further study to determine essentiality. It is anticipated that after such experimental study, at worst, the plant mass savings described in electronic supplementary material, table S4 will be completely offset by the mass cost of the nutrients and growth media. However, there are two reasons why plant mass savings may still exist. First, it should be possible to achieve greater mass savings and shorter manufacture periods than that described in electronic supplementary material, table S4 with tailored electrolysers, bioreactors and feeding processes. That is, a design optimization is needed: for instance, a higher volume electrolyser may weigh slightly more and allow for a slightly larger and heavier bioreactor, but this increase reduces the resultant process time, which correspondingly reduces required nutrient mass. Second, nutrient recycling is already an active research area, and both NASA and the US DOE are interested and invested in such research [95–99]. It is currently possible to recycle minerals of nitrogen, sulfur and phosphorus at between 80% and 100% efficiency [97], and data from the late 1990s also display substantial (greater than 65%) recycling efficiencies for potassium, calcium and manganese [96]. However, those early outcomes indicated the need for improvement in recycling copper, zinc and iron nutrients [96]. But iron, for example, may now be supplemented by extraction from the local Martian soil or lunar regolith.

#### 3.2.2. Nitrous oxide–hydrocarbon

There is a significant potential fuel benefit to producing nitrous oxide biologically on Mars, perhaps in combination with a hydrocarbon to achieve a mixture that has a high specific impulse. This benefit results from reducing the launch mass cost of complete nitrous oxide–hydrocarbon propellant delivery. However, as described in §S3, the overall mass and time costs render nitrous oxide–hydrocarbon biomanufacture impractical for now.

### 3.3. Synthetic biology improvements to biological propellant generation methods

It is expected that near-future space synthetic biology efforts on methanogenic bacteria will concentrate on minimizing nutrient use and improving bioreactor nutrient recycling percentages, rather than increasing productivity. This is due to the already high methane purity and production rates that have been previously reported in the literature. Because the analysis in §3.2.1 indicates that significant reductions of production-plant mass are possible and that a biomanufacture time that is less than half of Mars residence time can be realized, it is feasible to trade-off a portion of these mass and time savings (i.e. decrease methane volumetric output) for greater nutrient efficiency. This may require directed evolution experiments, where selection for high methane production is conducted, whereas the bacteria grow in reduced or minimal media and in conditions that emulate anticipated nutrient recycling processes.

Future solutions to the biological space production of nitrous oxide–hydrocarbon could include the following approaches. The first involves synthetic biology improvements to methanogenic bacteria [100–102] and cyanobacteria [103] (or the utilization of their relevant genes in other organisms) to enhance ethane production and bypass the acetate intermediate. The cited references describe bacteria that are capable of producing small quantities of ethane under various conditions: in the case of methanogenic bacteria, when incubated under hydrogen, exposed to halogenated hydrocarbons or stimulated by reduced, ethylated sulfur compounds, and in the case of cyanobacteria, when vanadium and iron nitrogenases reduce acetylene. As indicated by Lee *et al*. [104], the vanadium nitrogenase can yield ethane and propane by reducing carbon monoxide, and it has also been shown that remodelled nitrogenase can reduce carbon dioxide to methane and be coupled to the reduction of other substrates [105]. Thus, two possible synthetic biology investigations are the following: the reduction of carbon dioxide to ethane in cyanobacteria with the vanadium nitrogenase (which requires engineering for a different input), or the reduction of carbon dioxide to ethane in cyanobacteria with the remodelled nitrogenase (which requires engineering for a different output, possibly through coupling).

A second similar potential synthetic biology development involves increasing the cyanobacterial production of ethylene [106,107] with a carbon dioxide substrate from the current experimental maximum rate of 171 mg per litre per day in *Synechocystis* sp. PCC 6803 (when it employs the *efe* gene from *Pseudomonas syringae pv. Phaseolicola* [107]) by a few orders of magnitude, because ethylene is an alternative hydrocarbon that can be blended with nitrous oxide [108]. Lastly, synthetic biology refinements of glycogen-accumulating organisms (GAOs [109], see electronic supplementary material, §S3) to accomplish both nitrification when in oxygen as well as anaerobic denitrification to nitrous oxide are desirable.

## 4. Food production in space

### 4.1. Current food production method and cost

References [42] and [110] specify a rate of 1.83 kg of required food mass per crew member per day based on current ISS food container provisions, resulting in a total of 10 058 kg of similarly prepared food that needs to be shipped for the entire long-stay Mars mission. More accurate complete-meal ‘wet-food’ numbers can be obtained by scaling up the recent detailed mission results in [111] from 600 to 916 days. This process suggests that 10 403 kg of vegetarian food without packaging is necessary for the complete Mars voyage, with 5861 kg of this amount coming from local crops at a food cost of 4542 kg in shipped mass. An alternative varied or ‘mixed’ menu results in a total of 9537 kg of required food without packaging, of which 4542 kg is grown locally and the remaining 4995 kg of bulk commodities, minors and prepared foods constitute the shipped food cost. Thus, the expected wet-food cost of a Mars mission is between 4.5 and 5.0 T. A proportional estimate for a Moon mission puts the cost at between 0.62 and 0.68 T of shipped, unpackaged wet-food.

Over the course of 706 days of Mars stay and return travel to Earth for six crew members, the approximate mass cost can be calculated to be 3501 kg of vegetarian unpackaged wet-food, or 3850 kg of mixed unpackaged wet-food. If the switch is made during these 706 days to dry-food biomass, all of which would be produced on Mars during the 496 day residence on the planet, only 0.617 kg of completely dehydrated biomass is required to be consumed by a crew member per day [42]. This implies that 2614 kg of nutritious biomass has to be generated on Mars and analysed for shipped mass cost. A similar switch during a four-person lunar mission upon Moon arrival necessitates the generation of 453 kg of nutritious biomass, the cost of which must be compared with costs of either 607 kg of vegetarian unpackaged wet-food, or 667 kg of mixed unpackaged wet-food.

### 4.2. Biological food production method and cost

The chief food- and glucose-producing biological options are autotrophs like photosynthetic bacteria and plants. Fortunately, these organisms primarily require only those elemental and compound resources that are readily available on lunar and Martian missions. The case for using photosynthetic bacteria as an extraterrestrial food production option is effectively made in the comprehensive papers [31,112], where the nutritionally rich *Arthrospira platensis* and *Arthrospira maxima* (together forming the supplement *Spirulina*) are identified as a competitive option to plant-based space farming. Here, we compute the potential savings espoused by these works.

**Figure 6.**
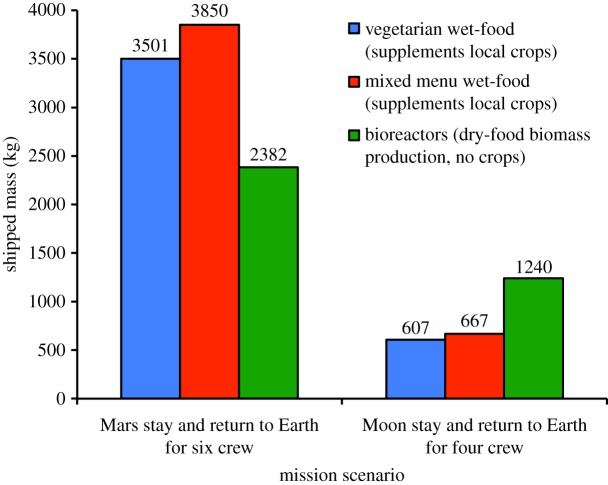
Current *Spirulina* biomass production techniques can represent a mass savings for food production on a Martian mission but not on a lunar mission.

*Spirulina* is currently experimentally produced at about 1 g l^−1^ d^−1^ of dry weight biomass in a closed bioreactor [31]. With three 2000 l bioreactors each having a working volume of 1757 l, it is possible to produce 5.271 kg of *Spirulina* per day. Over 496 days of Mars residence, this rate results in the production of the entire 2614 kg amount of nutritious *Spirulina* biomass that must be generated for use by six crew members during their stay on that planet and on the return journey to the Earth (§3.2.1). Assuming nutrient extraction from the Martian environs as well as complete nutrient recycling (see §3.2.1), the mass cost of the food-producing bioreactors is 2382 kg (figure 6). This computes to a saving of 32% over the mass cost of vegetarian unpackaged wet-food, and 38% over the mass cost of mixed-menu unpackaged wet-food. The bioreactors will draw 12.687 W of power [88] and require a volume of 14.844 m^3^ (table 4).

A single 2000 l bioreactor producing 2 kg of *Spirulina* per day generates 360 kg of nutritious biomass over 180 days, which is 79% of the amount that is required for a four-person lunar stay and return voyage. However, the mass cost of this bioreactor, at 794 kg, exceeds the mass costs of wet-food provisions (between 607 and 667 kg, as per §4.1, the expected mass cost for 100% biomass production is 1240 kg; figure 6). This lack of mass reduction may be attributed to the time duration allotted to lunar food production. Assuming local nutrient extraction and nutrient recycling (again, a feasible assumption per §3.2.1), the current break-even bioreactor mass cost of 611 kg for a 1000 l bioreactor and a 50 l bioreactor results in the production of 189 kg of biomass over 180 days, or about 42% of the requisite amount. If a lunar habitat becomes continuously manned similar to the ISS, then it should be possible to realize the savings of scale as on a Martian mission, and astronauts will be able to subsist primarily on *Spirulina*.

### 4.3. Synthetic biology improvements to biological food production methods

The preceding lunar analysis indicates that space synthetic biology efforts in food production should target increases in the *Spirulina* biomass productivity rate, without which only a proof-of-concept demonstration of nutritious biomass generation for astronaut consumption on the Moon is advised. A productivity rate increase from 1 g l^−1^ d^−1^ is possible because of reports of 50–100% higher *Spirulina* volumetric yields when employing tailored conditions and reactors: 2.1 g l^−1^ d^−1^ on elevated plates and 1.5 g l^−1^ d^−1^ in tubular photobioreactors [113].

Of course, eating solely dehydrated biomass for 706 days during a Mars mission would be extremely trying for astronauts, given their evolving palatability preferences and their need for sufficient menu variety [114]. Hence, the motivation for synthetic biology techniques to not only improve the nutraceutical rate of cyanobacterial fixation of carbon dioxide, but also to enhance and diversify the flavours and textures of generated biomass, along the lines of [115]. These techniques are better than simpler food processing approaches because they also offer the opportunity to incorporate healthful bioactive peptides that can prevent cardiovascular disease, inflammation, cancer, etc. [116,117]. The goal of flavour and texture enhancement is reminiscent of efforts to produce ‘yeast meat’ in the 1960s with mycoprotein from *Fusarium venenatum* A3/5 that were so successful that current yield rates of 300–350 kg of biomass h^−1^ of *Quorn* are possible [118]. However, *Quorn* production is significantly less energy efficient than that of *Spirulina* [119]. Likewise, *in vitro* meat production has recently recaptured the public's imagination with the announcement of a synthetic burger made of cultured beef [120]. But, there is still a lot to be desired ‘with respect to taste, look, mouthfeel and nutritional value’, and also ‘fat content, protein composition and … larger fibres or full-thickness cuts of meat’ [120].

## 5. Biopolymer synthesis in space

### 5.1. Current three-dimensional printing media synthesis method and cost

Case studies for additive manufacturing in space include that of habitat construction. A crude indication of the cost of three-dimensional printing a habitat is available via [11], which estimates that 3.8 T of dry salts must be shipped to the Moon to be mixed with regolith containing 9.6% MgO and locally excavated water to make ‘ink’ to construct 6 m^3^ of habitat encompassing 40 m^3^ of volume. Once associated printer and construction support material is included, the total launch mass for printing a habitat increases to about 8 T [11]. Because the suggested minimum habitat volume is 20 m^3^ per crew member for missions lasting longer than four months [121], with a recommended habitat volume of 120 m^3^ per crew member for long-duration missions [121], the cost of three-dimensional printing a four-person habitat for a Moon mission computes to between 16 and 96 T of shipped mass, of which between 7.6 and 45.6 T is dry salts, i.e. printer feedstock. The respective habitat structure volume is between 12 and 72 m^3^. An approximately equal concentration of MgO in the Martian soil as the lunar regolith puts the shipped mass cost for a six-person Mars mission at between 24 and 144 T, of which between 11.4 and 68.4 T is dry salts. The respective habitat structure volume is between 18 and 108 m^3^.

### 5.2. Biopolymer synthesis method and cost

The results of the stereolithographic habitat construction process of Cesaretti *et al*. [11] can also be accomplished with a three-dimensional printer that extrudes plastic media using fused deposition modelling (FDM). A comparison of the different additive manufacturing methods and possible feedstocks is included in [49], which explains why FDM is preferred over stereolithography for space applications. To determine the existence of launch mass savings for three-dimensional printed habitats from biological processes, this section examines the extraterrestrial manufacture of feasible three-dimensional printer raw materials like polyhydroxyalkanoate (PHA) biopolymers, of which polyhydroxybutyrates (PHBs) are an example. A contrast of habitat structural properties when using either salt-MgO or biopolymers as printer media is beyond the scope of this paper.

The biomanufacture of PHAs is reviewed in [122–125], and Lu *et al*. [123] include a list of organisms known to synthesize PHAs. It has been reported that PHA levels in bacteria can be accumulated as high as 90% of the cell dry mass, with such accumulation typically acting as a carbon and energy storage mechanism in times of nutrient stress [122]. Thus, it is feasible to alter the biological space production of a commodity during times of minimal need of that commodity by stressing the organisms involved in the process so that they synthesize biopolymers instead. This twin-manufacture intent serves to reduce the bioreactor and feedstock mass costs that would otherwise be incurred with producing PHA separately. An example of an organism that is a candidate for the production of two commodities is the food-producing cyanobacterium of §4.2, *A. platensis*, which can currently accumulate PHB at up to 10% of the cell dry mass when starved of nitrogen [126]. In some cases, PHA synthesis occurs as an interim stage of the biomanufacture of another commodity, as for instance with the nitrous oxide-producing GAOs of electronic supplementary material, §S3: GAOs use acetate and consume their glycogen to synthesize PHA, which is then harnessed to replenish glycogen and produce nitrous oxide [109]. Interruption of nitrous-oxide production yields PHA, and this dual manufacture possibility enhances the promise of GAOs.

There exist organisms other than GAOs that take up acetate to produce PHA [127–130], accumulating as much as 89% of the cell dry mass within 7.6 h [130]. Because acetate bioproduction in space is time-consuming (electronic supplementary material, §S3), we are interested in organisms that can directly convert carbon dioxide into PHAs, such as those described in [131]. One such organism is the autotroph *C. necator*, also known as *Ralstonia eutropha*, which is well studied [132–135] and can accumulate the largest percentage of cell dry mass as PHB from a C_1_-type substrate [131]. A PHB production of rate of 1.55 g l^−1^ h^−1^, i.e. 37.2 g l^−1^ d^−1^, has been reported for the organism while it was supplied with hydrogen and tested in the presence of a low (6.9%) volume of oxygen [132].

**Figure 7.**
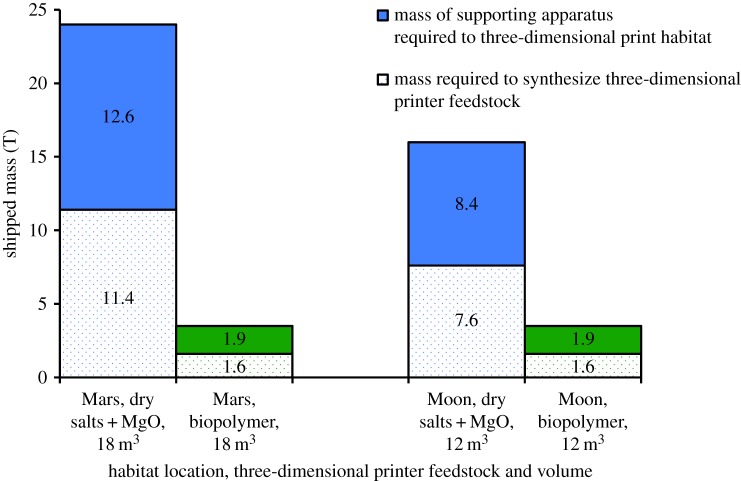
It may be significantly cheaper to three-dimensional print a habitat on both Mars and Moon missions by switching from stereolithographic additive manufacturing to biopolymer-based fused deposition modelling.

To produce 18 m^3^ of three-dimensional printed habitat structure on Mars encompassing a volume of 120 m^3^ for six crew, a current estimate of shipped mass cost is 24 T, of which 11.4 T is dry salts to mix with the MgO in the Martian soil for printer feedstock (§5.1). Substituting this mixture with PHB, which has a density of 1250 kg m^−3^, the required 18 m^3^ of habitat has a mass of 22 500 kg. Consider the following unoptimized biomanufacture scenario: two 2000 l bioreactors, each at a working volume of 1500 l, resulting in the production of 111.6 kg d^−1^ of PHB. In this scenario, 202 days are needed to synthesize 22 500 kg of PHB, an identical duration (41% of Mars stay) as that required for methane biomanufacture. The total empty mass of the bioreactors is 1.6 T (figure 7), an 86% savings over the 11.4 T of shipped dry salts. Drawn power can be calculated to be 7.221 W using [88], and the volumetric size of the bioreactors works out to be 9.896 m^3^ (table 4).

Because the PHB stoichiometry is [132]
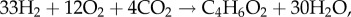
and the output H_2_O can be recycled and electrolysed, only 3H_2_ is required to be provided to sustain the reaction via the electrolysis of water extracted from soil that has been excavated and processed. This computes to a requirement of 7.84 kg of H_2_ per day, which is less than the maximum of 8.14 kg of H_2_ per day that can be produced with a single 6 kg electrolyser running at a flow rate of 2998 g h^−1^ (electronic supplementary material, table S4) that is part of a 1828 kg soil processing plant [84]. Hence, assuming local nutrient extraction and complete nutrient recycling (see §3.2.1), the shipped mass cost to generate material to three-dimensional print a habitat works out to about 3.5 T (figure 7), a savings of 85% on the original 24 T estimate.

A similar substitution of PHB can be made to replace a mixture of dry salts and MgO from the lunar regolith for use in three-dimensional printing 12 m^3^ of lunar habitat structure volume. The Martian biomanufacture scenario can be reproduced nearly exactly for a test run: the two 2000 l bioreactors, each with working volumes of 1500 l and a total production of 111.6 kg d^−1^ of PHB, can synthesize the requisite 15 000 kg of PHB mass in about 134 days, or 74% of lunar residence time. The mass cost of these bioreactors represent a 79% reduction over the 7.6 T of dry salt printer feedstock previously deemed necessary. Including a regolith processing plant of similar size as that proposed for a Martian mission keeps the total mass reduction also at 79% when compared with the published estimated total shipped mass of 16 T. Again, the bioreactors would require 7.221 W of power [88] and 9.896 m^3^ of space (table 4).

Because the cell mass stoichiometry for exponentially growing *C. necator* cells is [132]
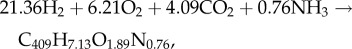
all necessary reactants are available during travel as a result of crew-generated wastes and hydrogen and oxygen stores (table 1). Thus, PHB generation is also feasible during voyage in case the need for three-dimensional printing arises. This suggests that the design and versatility of interlocking three-dimensional printed PHB blocks (e.g. to construct furniture) also requires investigation.

### 5.3. Synthetic biology improvements to biopolymer synthesis methods

It should be possible to improve the PHB accumulation percentage of *A. platensis* with synthetic biology approaches, because such techniques have allowed the cyanobacterium *Synechococcus* sp. PCC7002 to stably accumulate PHA at up to 52% of the cell dry mass when using the complementation of a cyanobacterial *recA* null mutation with the *Escherichia coli recA* gene on a plasmid expression system [136,137]. Synthetic biology efforts to combine the benefits of *C. necator* with the cyanobacterium *Synechocystis* sp. PCC 6803 of §3.3 have also been documented [138,139]. Such efforts are important because they lend credence to the idea of one organism that is capable of producing multiple commodities: fuel, biopolymers and as we shall see, possibly pharmaceuticals as well.

## 6. Pharmaceuticals in space

### 6.1. Current pharmaceutical usage and cost

At present, workarounds for accelerated pharmaceutical expiry involve the launch of one or more small unmanned spacecraft to restock crew supplies as needed. However, the practicality of these missions hinges on the location of a supply depot, conducive launch opportunities, travel time, etc., with corresponding implications on astronaut health in the event of a medical emergency. Thus, the cost of pharmaceutical shelf-life includes the cost of crew safety in addition to the cost of a resupply mission. The former is not easy to quantify but is regarded as paramount to reduce.

### 6.2. Synthetic biology approaches to on-demand pharmaceutical manufacture and their cost

**Figure 8.**
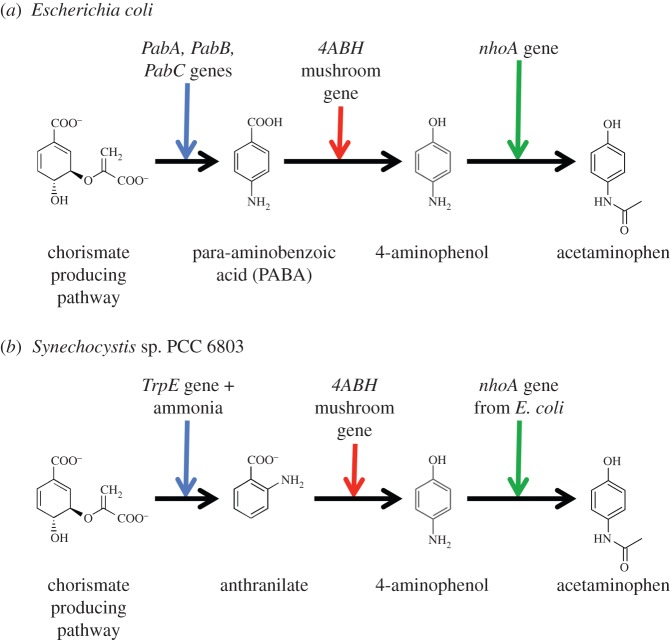
It is hypothesized that acetaminophen production in space is possible despite resource availability constraints if an analogous synthetic biology approach to one that was recently successfully experimentally tested is used. (*a*) Current synthetic biology approach [150] to manufacturing acetaminophen. (*b*) Proposed synthetic biology approach to manufacturing acetaminophen.

The advantageous role of biotechnology in pharmaceutical manufacture is known [140], and examples include antibiotics [141–143], antibiotic alternatives [144–146] such as bacteriophages [147,148], and even cancer chemotherapy [149]. Biotechnology can also facilitate the synthesis of antibiotics such as doxycycline and tetracycline from an acetate substrate, albeit with involved steps [141]. To tackle the typically complicated substrate sequence and pathway identification problem to desirable chemical targets, a computerized suite of tools has been recently developed [150]. This platform also suggests the amount of synthetic biological engineering that is necessary to accomplish the manufacture of the target. For instance, Anderson *et al*. [150] explain how acetaminophen can be produced by modifying the chorismate pathway (figure 8*a*), wherein *p*-aminobenzoic acid that is made from chorismate is transformed into 4-aminophenol using the *4ABH* gene taken from a mushroom, and this 4-aminophenol can then be used as a substrate to produce acetaminophen with the *E. coli nhoA* gene. The proposed pathway was successfully experimentally tested in an engineered strain of *E. coli* [150]. Unfortunately, this strain is not directly suitable for space application, on account of the reduced availability of the resources that the strain uses.

However, it should be possible to implement the above path to acetaminophen by harnessing a similar chorismate pathway in an autotroph of electronic supplementary material, table S1 that uses readily available inputs on space missions. The cyanobacterium *Synechocystis* sp. PCC 6803, for example, has the genes for chorismate synthase [151] and the *trpG* and *trpE* genes [152] that are homologues of the respective *E. coli pabA* and *pabB* genes [153], allowing it to generate an analogue [153] of *p*-aminobenzoic acid called anthranilate. (Note that *trpE* alone without *trpG* can also catalyse the formation of anthranilate, and is preferable because it uses the readily available resource of ammonia instead of glutamine.) Moreover, anthranilate can also be acted upon by the *4ABH* gene and 4-aminobenzoate hydroxylase [154]. It is therefore plausible that acetaminophen can be synthesized by *Synechocystis* sp. PCC 6803 after the insertion of the *4ABH* and *nhoA* genes (figure 8*b*), and testing of this hypothesis in the near-future is anticipated.

As Ungerer *et al*. [107] indicate, unnatural gene insertions into *Synechocystis* sp. PCC 6803 and subsequent organism optimization can allow for a substantial rate of production of a desired target. It is possible to estimate the volumetric yield of acetaminophen biomanufacture in *Synechocystis* sp. PCC 6803 with the data in [107]: an equivalent carbon output for the reported productivity rate of 171 mg l^−1^ d^−1^ (6 mmol l^−1^ d^−1^) of ethylene occurs when 1.5 mmol l^−1^ d^−1^ of acetaminophen is produced, i.e. 230 mg l^−1^ d^−1^. This yield is sufficient, because pharmaceuticals require low concentrations of an active ingredient, for example, a single tablet dose of acetaminophen is 325 mg. Hence, even accounting for losses, only a few days of acetaminophen manufacture starting from protected, inactive bacteria (that can survive in space and be tested for performance as explained in §2.2) will be required to replenish stocks of the pharmaceutical that have expired early because of space radiation. This renders crews independent from resupply spacecraft that could take at least 210 days to arrive after Earth launch. Moreover, the anticipated productivity rate should render the pharmaceutical production apparatus eminently portable and suitable for use on long voyages, because the required bioreactor working volumes need only be around 2 l or less. According to Stoker [88], this volume in a 3 l bioreactor would draw about 43.7 mW.

## 7. Summary

**Figure 9.**
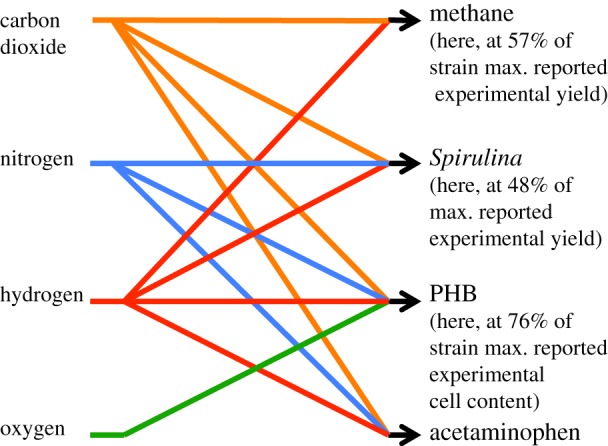
The solution biology map that uses the organisms in table 5.

**Figure 10.**
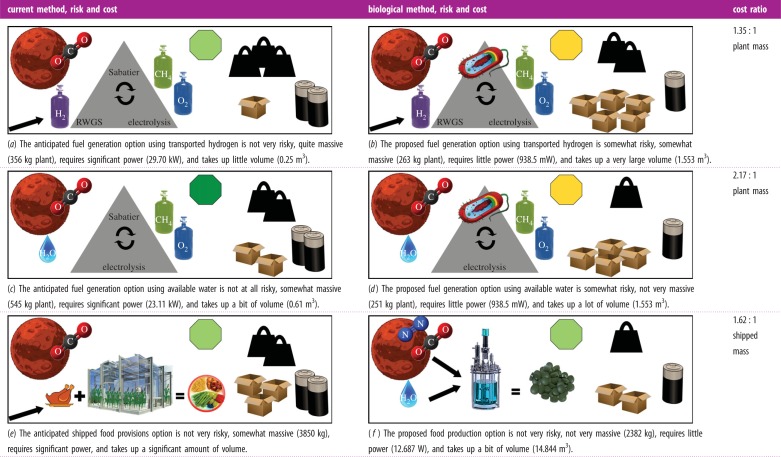

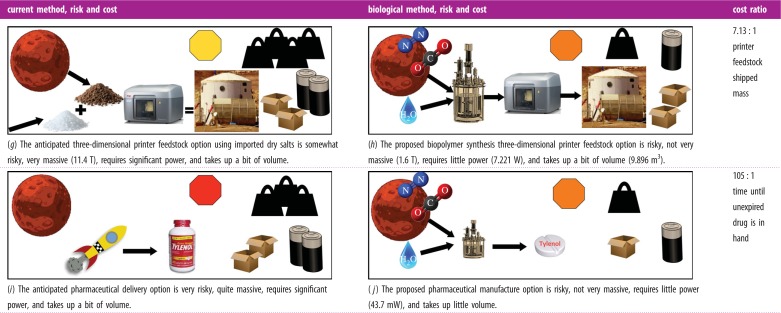
Comparative schematic analysis of the proposed solution to the Mars biology mapping problem.

The solution to the design problem that was posed at the start of this paper can be summarized by table 5 and figures 9 and 10. A few benign assumptions are required for this solution, and these assumptions have been verified in so far as currently possible. The assumptions include that of complete nutrient extraction and recycling from the Martian soil or lunar regolith; similar productivities in bioreactors on the Earth, Mars and the Moon and an engineered acetaminophen productivity that is of the same order as an estimate derived from engineered ethylene productivity when the organism chassis and the number of gene insertions are the same. The high-impact synthetic biology molecules and processes for space exploration discussed earlier in this work are listed in the following sections.

**Table 5.** Solution to the biology mapping problem.

inputs	biology mapping	outputs
CO_2_, H_2_	*Methanobacterium thermoautotrophicum*	CH_4_–O_2_ propellant
CO_2_, H_2_, N_2_	glycogen-accumulating organisms, engineered *Synechocystis*	N_2_O–hydrocarbon propellant
CO_2_, H_2_, N_2_	*Arthrospira platensis*, *Arthrospira maxima*	*Spirulina* food
CO_2_, H_2_, N_2_, O_2_	*Cupriavidus necator*	polyhydroxybutyrate biopolymer
CO_2_, H_2_, N_2_	engineered *Synechocystis*	acetaminophen pharmaceutical

### 7.1. List of important space synthetic biology organisms

— *Acetobacterium woodii*
— strain *KN-15* of *Methanobacterium*
— *M. thermoautotrophicum*
— deep marine subsurface archaea and bacteria that produce ethane and propane
— GAO
— methanogenic bacteria and cyanobacteria that produce ethane
— *Synechocystis* sp. PCC 6803 with the *efe* gene from *Pseudomonas* *syringae* pv. *Phaseolicola*
— *A. platensis*
— *A. maxima*
— *Fusarium venenatum* A3/5
— *Synechococcus* sp. PCC7002 using the complementation of a cyanobacterial *recA* null mutation with the *E. coli* *recA* gene on a plasmid expression system.
— *C. necator* H16, also known as *R. eutropha* H16
— *Synechocystis* sp. PCC 6803 with the PHA biosynthetic genes of *C. necator* or with overexpressed *sigE*
— *Synechocystis* sp. PCC 6803 with the *4ABH* and *nhoA* genes.

### 7.2. List of important space synthetic biology processes

— methanogenesis and electrolysis
— ethanogenesis and denitrification
— ethylenogenesis and denitrification
— stress response and PHA accumulation

### 7.3. List of important space synthetic biology products

— acetate
— methane
— ethane
— propane
— nitrous oxide
— ethylene
— acetylene
— *Spirulina*
— cultured meat
— PHB
— acetaminophen

## 8. Conclusion and future work

This paper has reviewed existing biological processes to demonstrate that they constitute a competitive yet non-traditional technology that is capable of processing volatiles and waste resources readily available on lunar and Martian space missions in a way that, when compared with anticipated non-biological approaches, reduces the launch mass of propellants, food and raw material for three-dimensional printing, and also overcomes the decreased product shelf-life of a common therapeutic. Specifically, it is determined that for Martian missions: 205 days of high-quality methane and oxygen Mars bioproduction with *M. thermoautotrophicum* can reduce the mass of a Martian fuel-manufacture plant by 56%; 496 days of biomass generation with *A. platensis* and *A. maxima* on Mars can decrease the shipped wet-food mixed-menu mass for a Mars stay and a one-way voyage by 38%; 202 days of Mars PHB synthesis with *C. necator* can lower the shipped mass to three-dimensional print a 120 m^3^ six-person habitat by 85%; and a few days of acetaminophen production with engineered *Synechocystis* sp. PCC 6803 can completely replenish expired or irradiated stocks of the pharmaceutical, thereby providing independence from unmanned resupply spacecraft that take up to 210 days to arrive. Analogous results are included for lunar missions.

The results suggest that future synthetic biology and technological efforts should focus on improving bioreactor nutrient recycling percentages, enhancing bioproduction of alternative nitrous oxide fuels, bettering flavours of *Spirulina*, testing interlocking three-dimensional printed PHB blocks in habitat and furniture construction, and increasing biosynthesis efficiencies of desired outputs, including pharmaceuticals. Because there also exist organisms that can leverage carbon monoxide on a large scale, the product yields that can be achieved by converting carbon dioxide to a biological feedstock of carbon monoxide remain to be explored. Perhaps this exploration can be achieved with a more formal constructed model, which consists of plug-and-play modules of biological pathways and processes that can be interchanged to predict outputs and their levels and help determine overall process efficiencies when including reactor sizing, power requirements, etc. The benefits of using communities and symbiotic organisms instead of single organism species is another avenue of future work. Next steps should also include testing this paper's biological designs in simulated lunar and Martian atmospheric conditions to explore the effect of different gas partial pressures and percentage concentrations on organism fitness, determine the adverse effects of long-term radiation on production performance and develop ways to efficiently extract newly synthesized products from organisms while in an extraterrestrial environment.

## Supplementary Material

(Toward_SynBio_ISRU_Supp.pdf) Toward Synthetic Biological Approaches to Resource Utilization on Space Missions: Supplementary Materials
