# Supplementary material for: Towards synthetic biological approaches to resource utilization on space missions
Source: J R Soc Interface. 2015 Jan 6;12(102):20140715. doi: 10.1098/rsif.2014.0715 (PMC4277073; doi:10.1098/rsif.2014.0715)
Supplement: (Toward_SynBio_ISRU_Supp.pdf) Toward Synthetic Biological Approaches to Resource Utilization on Space Missions: Supplementary Materials [file rsif20140715supp1.pdf]

Toward Synthetic Biological Approaches to Resource Utilization on Space Missions

Supplementary Materials

Amor A. Menezes[1], John Cumbers[2], John A. Hogan[3], and Adam P. Arkin[4]

1. California Institute for Quantitative Biosciences, University of California, Berkeley, 2151 Berkeley Way, Berkeley, CA 94704-5230, USA; amenezes@berkeley.edu
2. NASA Ames Space Portal, NASA Ames Research Center, MS 555-2, Moffett Field, CA 94035, USA; john.cumbers@nasa.gov
3. Bioengineering Branch, NASA Ames Research Center, MS 239-15, Moffett Field, CA 94035, USA; john.a.hogan@nasa.gov
4. E.O. Lawrence Berkeley National Laboratory, 1 Cyclotron Road, MS 955-512L, Berkeley, CA 94720, USA; and Department of Bioengineering, University of California, Berkeley, CA 94720, USA; aparkin@lbl.gov

S1 Tabular Supporting Information

Table S1 summarizes the mechanisms of action and the outputs produced by organisms that take in carbon dioxide, as detailed by [1]. Table S2 provides a similar summary of organisms that utilize and produce various nitrogen compounds; these organisms also play a role in the microbial nitrogen cycle [2–6].

Table S3 details the methane-oxygen propellant production mechanisms and mass requirements for each of the Mars scenarios described in [7]. The associated mass costs for two different production options of a similar fuel mix for a lunar module ascent are also listed in Table S3.

Table S4 summarizes the Mars ISRU mass cost of strain *KN-15* of *Methanobacterium* and strain Marburg of *Methanobacterium thermoautotrophicum* when a flow rate constraint of 3,000 g of water/h is imposed by incorporating a 6 kg electrolyzer that was recently validated in [8]. Table S5 provides a similar summary of the Moon ISRU mass cost, based on complete carbon dioxide extraction from lunar regolith that has been excavated by a 108 kg excavator (scaled up from [9]).

Table S6 presents the unoptimized mass cost of nutrients and growth media, when extrapolated from the literature requirements, for Mars-based methane bioproduction over the anticipated manufacture period of 205 days.

Table S1: Summary of CO_2 Fixation in Autotrophs [1]

Product	Organisms	Mechanism	Energetic Cost
Glyceraldehyde 3-phosphate, which is useful for terpenoid biosynthesis [10].	Plants, algae, cyanobacteria, many aerobic or facultative aerobic proteobacteria belonging to the alpha, beta, and gamma subgroups, *Sulfobacillus* spp., iron and sulfur-oxidizing members of the firmicutes, some mycobacteria, green nonsulfur bacteria of the genus *Oscillochloris* (phylum *Chloroflexi*), and (anaerobic) photoheterotrophic growth of some purple bacteria (e.g., *Rhodobacter*, *Rhodospirillum*, and *Rhodopseudomonas*).	Reductive Pentose Phosphate (Calvin-Benson) Cycle, with key enzyme RubisCO.	Nine ATP equivalents and six NADPHs are required to synthesize one glyceraldehyde-3-phosphate molecule.
Acetyl-CoA, which must be converted to other intermediates of the carbon metabolism: pyruvate, phosphoenolpyruvate, oxaloacetate, and 2-oxoglutarate. It is a useful starting point for a number of high value chemicals and biofuels [11].	The green sulfur bacterium *Chlorobium limicola* (*Chlorobi*), anaerobic or microaerobic members of *Aquificae*, *Proteobacteria* (especially of the delta and epsilon subdivisions), and *Nitrospirae* (e.g., *Nitrospira* and *Leptospirillum*).	Reductive Citric Acid (Arnon-Buchanan) Cycle, which reverses the reactions of the oxidative citric acid cycle (Krebs cycle) and forms acetyl-CoA from two CO_2s.	In *Chlorobium*, two ATP equivalents are required to form pyruvate, and three additional ATPs are required to convert it to triose phosphates.
Acetyl-CoA, which can be used to generate acetate or methane in the process of energy conservation, and also used for the assimilation of a variety of C_1 compounds like carbon monoxide, formaldehyde, methanol, methylamine, methylmercaptane, and methyl groups of aromatic *O*-methyl ethers/esters.	Prokaryotes that live close to the thermodynamic limit such as acetogenic bacteria and methanogenic archaea, anaerobic ammonia-oxidizing planctomycetes, sulfate-reducing bacteria (*Desulfobacterium* sp., *Deltaproteobacteria*), and autotrophic *Archaeoglobales* (*Euryarchaeota*) growing by means of anaerobic respiration.	Reductive Acetyl-CoA (Wood-Ljungdahl) Pathway, which requires strict anoxic conditions and has high energetic efficiency.	In methanogens, no additional ATP input is required, but in bacteria, an additional ATP equivalent is required.
Acetyl-CoA, which is used to synthesize pyruvate and then other central precursor molecules.	Mostly anaerobic autotrophic representatives of *Thermoproteales* and *Desulfurococcales*, and a facultative aerobe of the *Desulfurococcales*, *Pyrolobus fumarii* at very low O_2 concentrations.	Dicarboxylate / 4-Hydroxybutyrate Cycle, which is anaerobic.	Five ATP equivalents are required to synthesize one pyruvate, and three additional ATP equivalents are required to synthesize one triose phosphate from pyruvate.
Acetyl-CoA, which is used to produce succinyl-CoA, which is then oxidatively converted to oxaloacetate, pyruvate and phosphoenolpyruvate.	(Micro)Aerobic *Sulfolobales*, and the mesophilic aerobic “marine group I” *Archaea* (“*Thaumarchaeota*”).	3-Hydroxypropionate / 4-Hydroxybutyrate Cycle, which is aerobic.	Nine ATP equivalents are required to generate three molecules of pyrophosphate, and three additional ATP equivalents are required to synthesize one triose phosphate from pyruvate.
Glyoxylate, which is converted to the cellular building blocks in a second cycle. The bi-cycle allows coassimilation of fermentation products such as acetate, propionate, and succinate, or 3-hydroxypropionate, an intermediate in the metabolism of the osmoprotectant dimethylsulfoniopropionate.	The green non-sulfur phototrophs of the *Chloroflexaceae* family, which grow preferentially under photoheterotrophic conditions. The only autotrophic representative of this family found so far is *Chloroflexus aurantiacus*.	3-Hydroxypropionate (Fuchs-Holo) Cycle, which has high efficiency carboxylases compared to RubisCO.	Seven ATP equivalents are required for pyruvate synthesis, and an additional three ATPs are required for triose phosphate.

Table S2: Selections from the Microbial N_2 Cycle [2–6]

Product	Organisms	Mechanism
Ammonia	*Rhizobium*, *Frankia*, *Azotobacter*, *Beijerinckia*, *Azospirillum*, *Acetobacter*, cyanobacteria, *Anabaena*, *Clostridium*	Biological Nitrogen Fixation
Nitrite	Ammonia-Oxidizing Bacteria (AOB), e.g., *Nitrosomonas*, *Nitrosococcus*, and Ammonia-Oxidizing Archaea (AOA)	Aerobic Ammonia Oxidation, with CO_2 as carbon source.
Nitrate	Nitrite-Oxidizing Bacteria (NOB), e.g., *Nitrobacter*, *Nitrospira*	Aerobic Nitrite Oxidation, with CO_2 as carbon source
Nitrite, Nitric oxide, Nitrous oxide, Nitrogen	*Pseudomonas*, *Bacillus*, *Thiobacillus*, *Propionibacterium*, glycogen-accumulating organisms	Denitrification in low-oxygen or anaerobic conditions

Table S3: Mass Cost of Current Mars and Moon Ascent Propellant Production Approaches

Name	Production Option [7, 12]	Production Mechanism [7, 13]	Mass Delivered [T] [7, 13]
Mars<br>Bring Methane	24,891 kg of O_2, 133 kg of N_2/Ar, bring 399 kg of H_2, bring 6,567 kg of CH_4	Reverse Water Gas Shift<br>$2CO_2 + 2H_2 \rightarrow 2CO + 2H_2O$<br>followed by water electrolysis<br>$2H_2O \rightarrow 2H_2 + O_2$	7.5
Mars<br>Bring Hydrogen	24,891 kg of O_2, 133 kg of N_2/Ar, 6,567 kg of CH_4, bring 2,069 kg of H_2	Sabatier<br>$CO_2 + 4H_2 \rightarrow CH_4 + 2H_2O$<br>and Reverse Water Gas Shift<br>$2CO_2 + 2H_2 \rightarrow 2CO + 2H_2O$<br>with water electrolysis<br>$2H_2O \rightarrow 2H_2 + O_2$	3.3
Mars<br>Find Water	24,891 kg of O_2, 133 kg of N_2/Ar, 6,567 kg of CH_4, excavate 16,788 kg of H_2O	Sabatier<br>$CO_2 + 4H_2 \rightarrow CH_4 + 2H_2O$<br>with water electrolysis<br>$2H_2O \rightarrow 2H_2 + O_2$	2.7 with 3wt% H_2O in soil,<br>2.0 with 8wt% H_2O in soil
Moon<br>Bring Hydrogen	7,600 kg of O_2, 2,160 kg of CH_4, bring 1,501 kg of H_2, bring 1,617 kg of C in plastic and packaging trash	Hydrogen reduction of FeO in a 10T capacity O_2 plant followed by water electrolysis, pyrolysis of plastic and packaging trash with the excess H_2 for CH_4	4.1 with 5wt% FeO in regolith (1.501 of H_2 + 1.617 C trash + 0.942 O_2 plant), 3.7 with 14wt% FeO in regolith (1.501 of H_2 + 1.617 C trash + 0.563 O_2 plant)
Moon<br>Find Water	7,600 kg of O_2, 2,160 kg of CH_4, bring 1,617 kg of C in plastic and packaging trash, excavate 8,588 kg of H_2O	Water electrolysis in a 10T capacity O_2 plant, pyrolysis of plastic and packaging trash with the obtained H_2 for CH_4	1.8 (1.617 C trash + 0.190 O_2 plant)

Table S4: Plant Mass Cost and Savings of Proposed Mars Ascent Methane Propellant Production Approaches

Name	Production Option	Production Mechanism	Mass and Time Summary
Mars<br>Bring Hydrogen<br>High Vol. Prod.<br>Low Gas Conc.	24,891 kg of O_2, 133 kg of N_2/Ar, 6,567 kg of CH_4, bring 1,651 kg of H_2	*Methanobacterium* strain *KN-15*<br>65 L volume in 100 L bioreactor<br>32 kg of biosynthesized CH_4/day<br>2,998 g of flowing H_2O/h<br>64 kg of electrolyzed O_2/day<br><br>and Reverse Water Gas Shift<br>11,792 kg additional total O_2 after separate electrolysis	Current Plant: 356 kg [7]<br>Proposed Plant: 217 kg<br>(193 kg bioreactor estimate + 6 kg electrolysis unit × 2 + 12 kg RWGS unit [14])<br>**Plant Reduction: 39%**<br>CH_4 Completion: 205 days<br>O_2 Completion: 205 days<br>(390 days without RWGS)<br>Current H_2: 2,069 kg [7]<br>Proposed H_2: 1,651 kg<br>**H_2 Reduction: 20%**
Mars<br>Bring Hydrogen<br>Low Vol. Prod.<br>High Gas Conc.	24,891 kg of O_2, 133 kg of N_2/Ar, 6,567 kg of CH_4, bring 1,651 kg of H_2	*M. thermoautotrophicum*<br>157 L volume in 200 L bioreactor<br>32 kg of biosynthesized CH_4/day<br>2,998 g of flowing H_2O/h<br>64 kg of electrolyzed O_2/day<br><br>and Reverse Water Gas Shift<br>11,792 kg additional total O_2 after separate electrolysis	Current Plant: 356 kg [7]<br>Proposed Plant: 263 kg<br>(239 kg bioreactor estimate + 6 kg electrolysis unit × 2 + 12 kg RWGS unit [14])<br>**Plant Reduction: 26%**<br>CH_4 Completion: 205 days<br>O_2 Completion: 205 days<br>(390 days without RWGS)<br>Current H_2: 2,069 kg [7]<br>Proposed H_2: 1,651 kg<br>**H_2 Reduction: 20%**
Mars<br>Find 3wt% H_2O<br>High Vol. Prod.<br>Low Gas Conc.	24,891 kg of O_2, 133 kg of N_2/Ar, 6,567 kg of CH_4, excavate 14,849 kg of H_2O (includes bioreactor working volume and solvent for an hour of nutrient flow, assuming recycling)	*Methanobacterium* strain *KN-15*<br>65 L volume in 100 L bioreactor<br>32 kg of biosynthesized CH_4/day<br>2,998 g of flowing H_2O/h<br>64 kg of electrolyzed O_2/day<br><br>Separate electrolysis of the excavated H_2O for H_2 also yields 13,108 kg O_2	Current Plant: 545 kg [7]<br>(527 kg if 8wt% H_2O [7])<br>Proposed Plant: 205 kg<br>(193 kg bioreactor est. + 2×6 kg electrolysis units)<br>**Plant Reduction: 62%**<br>**(61% for 8wt% H_2O)**<br>CH_4 Completion: 205 days<br>O_2 Completion: 205 days<br>Current H_2O: 16,788 kg [7]<br>Proposed H_2O: 14,760 kg<br>**H_2O Reduction: 12%**
Mars<br>Find 3wt% H_2O<br>Low Vol. Prod.<br>High Gas Conc.	24,891 kg of O_2, 133 kg of N_2/Ar, 6,567 kg of CH_4, excavate 14,938 kg of H_2O (includes bioreactor working volume and solvent for an hour of nutrient flow, assuming recycling)	*M. thermoautotrophicum*<br>157 L volume in 200 L bioreactor<br>32 kg of biosynthesized CH_4/day<br>2,998 g of flowing H_2O/h<br>64 kg of electrolyzed O_2/day<br><br>Separate electrolysis of the excavated H_2O for H_2 also yields 13,108 kg O_2	Current Plant: 545 kg [7]<br>(527 kg if 8wt% H_2O [7])<br>Proposed Plant: 251 kg<br>(239 kg bioreactor est. + 2×6 kg electrolysis units)<br>**Plant Reduction: 56%**<br>**(55% for 8wt% H_2O)**<br>CH_4 Completion: 205 days<br>O_2 Completion: 205 days<br>Current H_2O: 16,788 kg [7]<br>Proposed H_2O: 14,760 kg<br>**H_2O Reduction: 12%**

Table S5: Plant Mass Cost and Savings of Proposed Moon Ascent Methane Propellant Production Approaches

Name	Production Option	Production Mechanism	Mass and Time Summary
Moon<br>Bring Hydrogen<br>High Vol. Prod.<br>Low Gas Conc.	7,600 kg of O_2, 2,160 kg of CH_4, bring 958 kg of H_2, excavate 48,000 kg of regolith/day	*Methanobacterium* strain *KN-15*<br>43 L volume in 50 L bioreactor<br>21 kg of biosynthesized CH_4/day<br>1,965 g of flowing H_2O/h<br>42 kg of electrolyzed O_2/day<br>(equivalent to a plant producing 15T of O_2 annually)	Current: 2,559 kg or 2,180 kg at 5wt% FeO or 14wt% FeO in regolith, respectively [13]<br>(1,617 kg of C trash + 942 kg or 563 kg, respectively, for 10T O_2 plant)<br>Proposed: 331 kg<br>(165 kg bioreactor est. + 6 kg electrolysis unit + 80 kg excavator $\times$ 2)<br>**Reduction: 87%**<br>**(85% for 14wt% FeO case)**<br>CH_4 Completion: 103 days<br>O_2 Completion: 181 days (less if excavated water also electrolyzed)<br>Current H_2: 1,501 kg [13]<br>Proposed H_2: 958 kg<br>**H_2 Reduction: 36%**
Moon<br>Bring Hydrogen<br>Low Vol. Prod.<br>High Gas Conc.	7,600 kg of O_2, 2,160 kg of CH_4, bring 958 kg of H_2, excavate 48,000 kg of regolith/day	*M. thermoautotrophicum*<br>103 L volume in 200 L bioreactor<br>21 kg of biosynthesized CH_4/day<br>1,965 g of flowing H_2O/h<br>42 kg of electrolyzed O_2/day<br>(equivalent to a plant producing 15T of O_2 annually)	Current: 2,559 kg or 2,180 kg at 5wt% FeO or 14wt% FeO in regolith, respectively [13]<br>(1,617 kg of C trash + 942 kg or 563 kg, respectively, for 10T O_2 plant)<br>Proposed: 405 kg<br>(239 kg bioreactor est. + 6 kg electrolysis unit + 80 kg excavator $\times$ 2)<br>**Reduction: 84%**<br>**(81% for 14wt% FeO case)**<br>CH_4 Completion: 103 days<br>O_2 Completion: 181 days (less if excavated water also electrolyzed)<br>Current H_2: 1,501 kg [13]<br>Proposed H_2: 958 kg<br>**H_2 Reduction: 36%**
Moon<br>Find Water<br>High Vol. Prod.<br>Low Gas Conc.	7,600 kg of O_2, 2,160 kg of CH_4, excavate 4,911 kg of H_2O from 32,400 kg of regolith/day (includes bioreactor working volume and solvent for an hour of nutrient flow, assuming recycling)	*Methanobacterium* strain *KN-15*<br>29 L volume in 50 L bioreactor<br>14 kg of biosynthesized CH_4/day<br>1,326 g of flowing H_2O/h<br>28 kg of electrolyzed O_2/day<br><br>Separate electrolysis of the excavated H_2O in the regolith yields the remaining 3,275 kg O_2	Current: 1,807 kg [13]<br>(1,617 kg of C trash + 190 kg for 10T O_2 plant)<br>Proposed: 285 kg<br>(165 kg bioreactor est. + 2$\times$6 kg electrolysis units + 108 kg excavator)<br>**Reduction: 84%**<br>CH_4 Completion: 153 days<br>O_2 Completion: $<$153 days<br>Current H_2O: 8,588 kg [13]<br>Proposed H_2O: 4,851 kg<br>**H_2O Reduction: 44%**
Moon<br>Find Water<br>Low Vol. Prod.<br>High Gas Conc.	7,600 kg of O_2, 2,160 kg of CH_4, excavate 4,911 kg of H_2O from 32,400 kg of regolith/day (includes bioreactor working volume and solvent for an hour of nutrient flow, assuming recycling)	*M. thermoautotrophicum*<br>70 L volume in 100 L bioreactor<br>14 kg of biosynthesized CH_4/day<br>1,326 g of flowing H_2O/h<br>28 kg of electrolyzed O_2/day<br><br>Separate electrolysis of the excavated H_2O in the regolith yields the remaining 3,275 kg O_2	Current: 1,807 kg [13]<br>(1,617 kg of C trash + 190 kg for 10T O_2 plant)<br>Proposed: 313 kg<br>(193 kg bioreactor est. + 2$\times$6 kg electrolysis units + 108 kg excavator)<br>**Reduction: 83%**<br>CH_4 Completion: 153 days<br>O_2 Completion: $<$153 days<br>Current H_2O: 8,588 kg [13]<br>Proposed H_2O: 4,851 kg<br>**H_2O Reduction: 44%**

Table S6: Unoptimized Nutrient Mass Requirements for 205 Days of Mars Methane Propellant Production

Name	Production Mechanism	Nutrient Mass Summary
Mars<br>Bring Hydrogen<br>High Vol. Prod.<br>Low Gas Conc.	*Methanobacterium* strain *KN-15*<br>65 L volume in 100 L bioreactor	18.66–21.66 g/L nutrient weight concentration (since $NaHCO_3$ is between 5–8 g/L) [15, 16] ⇒<br>1.21–1.41 kg of initial media in 63.6–63.8 kg transported H_2O<br>0.3–0.4/h dilution rate [16] ⇒ 19.5–26 L/h media flow rate ⇒<br>1,791–2,862 kg of flowing media (includes 19.14–25.44 kg recycled flowing H_2O, 65 kg start media)
Mars<br>Bring Hydrogen<br>Low Vol. Prod.<br>High Gas Conc.	*M. thermoautotrophicum*<br>157 L volume in 200 L bioreactor	15.17 g/L nutrient weight concentration [17] ⇒<br>2.38 kg of initial media in 154.6 kg transported H_2O<br>0.15/h dilution rate [18] ⇒ 23.55 L/h media flow rate ⇒<br>1,938 kg of flowing media (includes 23.19 kg recycled flowing H_2O, 157 kg start media)
Mars<br>Find 3wt% H_2O<br>High Vol. Prod.<br>Low Gas Conc.	*Methanobacterium* strain *KN-15*<br>65 L volume in 100 L bioreactor	18.66–21.66 g/L nutrient weight concentration (since $NaHCO_3$ is between 5–8 g/L) [15, 16] ⇒<br>1.21–1.41 kg of initial media in 63.6–63.8 kg excavated H_2O<br>0.3–0.4/h dilution rate [16] ⇒ 19.5–26 L/h media flow rate ⇒<br>1,708–2,773 kg of flowing media (excludes 19.14–25.44 kg recycled flowing H_2O, start H_2O)
Mars<br>Find 3wt% H_2O<br>Low Vol. Prod.<br>High Gas Conc.	*M. thermoautotrophicum*<br>157 L volume in 200 L bioreactor	15.17 g/L nutrient weight concentration [17] ⇒<br>2.38 kg of initial media in 154.6 kg excavated H_2O<br>0.15/h dilution rate [18] ⇒ 23.55 L/h media flow rate ⇒<br>1,760 kg of flowing media (excludes 23.19 kg recycled flowing H_2O, start H_2O)

S2 Alternative Propellant and Generation Cost

There exists another rocket fuel candidate with specific impulse and density-specific impulse properties that are comparable to a methane-oxygen combination: the currently uncommon blended monopropellant, nitrous oxide-hydrocarbon, where the hydrocarbon is propane, ethane, ethylene, acetylene, etc. Nitrous oxide (N_2O) [19] has been analyzed for spacecraft propulsion as a monopropellant [20, 21] and as a blended monopropellant, for instance with propane (abbreviated as NOP) [22, 23], ammonia [24], and paraffin when mixed with oxygen (known as Nytrox) [25]. Nitrous oxide's appeal lies in its specific impulse properties (which are particularly high as a blended monopropellant), its lack of toxicity, and its relative ease of storage and handling. NASA has also studied nitrous oxide monopropellant rockets and their potential applications [26, 27], and is interested in proprietary blends of nitrous oxide and hydrocarbons for potential use as a Mars ascent vehicle propellant. These blends are collectively known as Nitrous Oxide Fuel Blends X (NOFBX) [28–31], where each fuel blend has X replaced by a two-digit number that designates the hydrocarbon used and its mixture ratio. These hydrocarbons include ethane, ethylene and acetylene [32].

The mass of a nitrous oxide-hydrocarbon blended monopropellant that is required to lift a Mars ascent vehicle to orbit can be determined as follows. Since the amount of thrust that is required for ascent is $\Delta V = 5.625$ km/s [7], and the specific impulse of the methane-oxygen propellant is $I_{sp} = 371$ s (at a nozzle expansion ratio of 200 and a chamber pressure of 68 atm) [33] with a fuel mass $m_f = 31,458$ kg (Table S3), the ideal rocket equation,

$$\Delta V = I_{sp} g \ln \frac{(m_1 + m_f)}{m_1},$$

where $g = 9.80665$ m/s^2 suggests that the mass of the Mars ascent vehicle, m_1, is about 8,518 kg. Using the ideal rocket equation again, along with a specific impulse of $I_{sp} = 325$ s taken from that of NOFBX [31] (which has a theoretical maximum I_{sp} of 345 s; NOP to vacuum has a comparable I_{sp} of 312 s [23]), the mass of a nitrous oxide-hydrocarbon blended monopropellant that is required for ascent computes to about 41,237 kg. It is anticipated that this entire required mass will be launched from Earth if this blended monopropellant is selected for Mars ascent, although the "potential for *in situ* top-off" has also been identified [30]. It is possible to convert carbon dioxide to ethylene with the electrochemical approach in [34].

S3 Biological Nitrous Oxide-Hydrocarbon Generation Methods and Costs

There is a significant potential fuel benefit to producing nitrous oxide biologically on Mars, perhaps in combination with a hydrocarbon to achieve a mixture that has a high specific impulse. This benefit results from reducing the launch mass cost of complete nitrous oxide-hydrocarbon propellant delivery. As stated in Section 2.4, we seek a manufacture process that utilizes a feedstock of biologically-produced acetate, which can be made with hydrogen electrolyzed from water that is extracted from the Martian soil. Such a process exists: the biological manufacture of ethane and propane from acetate can proceed with the organisms and conditions described in [35]; alternatively, ethane can be electrolyzed from acetate; and lastly, the so-called Glycogen Accumulating Organisms (GAOs) [36] take up acetate anaerobically to produce nitrous oxide via denitrification at an efficiency of either 90% with a nitrate nitrogen (NO_3-N) load, or 95% with a nitrite nitrogen (NO_2-N) load. The latter efficiency is the highest efficiency known [37]. Other biological nitrous oxide production mechanisms are also available [38], and the conditions for producing nitrous oxide are well-studied [39–47]. There is evidence that the idea of combining biologically-produced nitrous oxide with a rocket engine is actively being researched, for instance to power a wastewater treatment plant [48].

At an 8.0:10.0 mixture ratio of nitrous oxide to ethane [32], about 18,328 kg of the blend is nitrous oxide, and the remainder 22,909 kg is ethane. This ratio is selected from among the possibilities in [32] because it maximizes the percentage content of nitrous oxide, which has a greater acetate conversion efficiency than ethane. That is, the Kolbe electrolysis stoichiometry of

$$2CH_3COO^- \rightarrow CH_3CH_3 + 2CO_2 + 2e^-$$

requires 2 mol of acetate for every 1 mol of ethane produced, while GAOs require 17.20 mol of acetate for every 14.78 mol of N_2O produced with an NO_2-N load [36], and are therefore more efficient at acetate conversion. A total of 100,589 kg of acetate is required to produce the necessary ethane and nitrous oxide for the blended monopropellant. However, even with two 2,000 L bioreactors (that, together with a soil processing plant, weigh about as much as the shipped mass for the “Bring Hydrogen” case in Table S3), only 29.6 kg of acetate can be produced per day at the experimental autotrophic production rate of 7.4 g/L/day; hence, 3,398 days are required to biomanufacture the requisite acetate. Because additional reactor(s) are also needed to accomplish nitrification upstream of GAO nitrous oxide production, the overall mass and time costs render nitrous oxide-ethane biomanufacture impractical for now.

References

[1] Ivan A Berg. Ecological aspects of the distribution of different autotrophic CO_2 fixation pathways. *Applied and Environmental Microbiology*, 77(6):1925–1936, March 2011.

[2] Janet I Sprent. *The Ecology of the Nitrogen Cycle*. Cambridge University Press, 1987.

[3] Gary Stacey, Robert H Burris, and Harold J Evans, editors. *Biological Nitrogen Fixation*. Chapman and Hall, 1992.

[4] Mike S M Jetten. The microbial nitrogen cycle. *Environmental Microbiology*, 10(11):2903–2909, 2008.

[5] James W B Moir, editor. *Nitrogen Cycling in Bacteria*. Caister Academic Press, 2011.

[6] Discussion meeting issue on ‘The global nitrogen cycle in the twenty-first century’. *Philosophical Transactions of the Royal Society, B*, 368(1621), July 5 2013.

[7] Gerald B Sanders. In-situ resource utilization on Mars — update from DRA 5.0 study. In *Proceedings of the 48th AIAA Aerospace Sciences Meeting Including the New Horizons Forum and Aerospace Exposition*, number AIAA 2010-799, January 4–7 2010.

[8] Diane L Linne, James R Gaier, Joseph G Zoeckler, John S Kolacz, Robert S Wegeng, Scot D Rassat, and D Larry Clark. Demonstration of critical systems for propellant production on Mare for science and exploration missions. In *Proceedings of the 51st AIAA Aerospace Sciences Meeting Including the New Horizons Forum and Aerospace Exposition*, number AIAA 2013-0587, January 7–10 2013.

[9] Tricia Talbert and Mike Green. College team wins NASA lunar robot prize. *NASA Office of the Chief Technologist: Centennial Challenges*, October 10 2009. URL `http://www.nasa.gov/offices/oct/early_stage_innovation/centennial_challenges/cc_regolith_feature_first_prize.html`.

[10] Vikramaditya G Yadav, Marjan De Mey, Chin Giaw Lim, Parayil Kumaran Ajikumar, and Gregory Stephanopoulos. The future of metabolic engineering and synthetic biology: Towards a systematic practice. *Metabolic Engineering*, 14(3):233–241, May 2012.

[11] Pamela P Peralta-Yahya, Fuzhong Zhang, Stephen B Del Cardayre, and Jay D Keasling. Microbial engineering for the production of advanced biofuels. *Nature*, 488(7411):320–328, August 16 2012.

[12] Gerald B Sanders, William E Larson, Kurt R Sacksteder, and Carole A Mclemore. NASA In-Situ Resource Utilization (ISRU) project — development & implementation. In *Proceedings of the AIAA SPACE 2008 Conference & Exposition*, number AIAA 2008-7853, September 9–11 2008.

[13] Donald Rapp. *Use of Extraterrestrial Resources for Human Space Missions to Moon or Mars*. Springer-Praxis, Chichester, UK, 2013.

[14] Robert Zubrin, Tomoko Kito, and Brian Frankie. Report on the construction and operation of a Mars in situ propellant production unit utilizing the reverse water gas shift. In *34th AIAA/ASEE Joint Propulsion Conference*, number AIAA-98-3303, 1998.

[15] Norihiro Nishimura, Shinko Kitaura, Akio Mimura, and Yoshimasa Takahara. Growth of thermophilic methanogen KN-15 on H_2-CO_2 under batch and continuous conditions. *Journal of Fermentation and Bioengineering*, 72(4):280–284, 1991.

[16] Norihiro Nishimura, Shinko Kitaura, Akio Mimura, and Yoshimasa Takahara. Cultivation of thermophilic methanogen KN-15 on H_2-CO_2 under pressurized conditions. *Journal of Fermentation and Bioengineering*, 73(6):477–480, 1992.

[17] Marie-Laure Fardeau and Jean-Pierre Belaich. Energetics of the growth of *Methanococcus thermolithotrophicus*. *Archives of Microbiology*, 144(4):381–385, 1986.

[18] Jean-Paul Peillex, Marie-Laure Fardeau, and Jean-Pierre Belaich. Growth of *Methanobacterium thermoautotrophicum* on H_2-CO_2: High CH_4 productivities in continuous culture. *Biomass*, 21(4):315–321, 1990.

[19] T P Gaidei. Nitrous oxide: Properties, producing, grounds of manipulations, and fields of application. *Russian Journal of Applied Chemistry*, 82(9):1689–1705, 2009.

[20] Vadim Zakirov. Catalytic decomposition of nitrous oxide for spacecraft propulsion applications (phase 1). Technical Report SPC 99-4100, EOARD, 2000.

[21] Vadim Zakirov and Martin Sweeting. Nitrous oxide as a rocket propellant. *Acta Astronautica*, 48(5–12):353–362, 2001.

[22] Jason S Tyll and Roger Herdy. The nitrous oxide - propane rocket engine. Technical Report ADA393448, Defense Technical Information Center (DTIC) Document, 2001.

[23] Roger Herdy. Nitrous oxide / hydrocarbon fuel advanced chemical propulsion: DARPA contract overview. In *Proceedings of the Thermal and Fluids Analysis Workshop 2006*, number TFAWS-06-1026, August 7–11 2006.
[24] Jessy R Jones. Mission and micronozzle flow analysis of a high-temperature chemical propulsion system. Technical Report ADA405088, Defense Technical Information Center (DTIC) Document, 2002.
[25] Ashley A Chandler, Brian J Cantwell, G Scott Hubbard, and Arif Karabeyoglu. Feasibility of a single port hybrid propulsion system for a Mars ascent vehicle. *Acta Astronautica*, 69:1066–1072, 2011.
[26] Robert Zubrin. NASA SBIR 00-1 solicitation: Nitrous oxide-organic liquid monopropellant rocket, 2000. Proposal number 00-1 20.02-8728, agency tracking number NASA 1273, award ID 51838.
[27] Robert Zubrin. NASA SBIR 01-1 solicitation: Nitrous oxide monopropellant rocket, 2001. Proposal number H4.01-9693, agency tracking number NASA 012306, award ID 56433.
[28] R Taylor. Safety and performance advantages of Nitrous Oxide Fuel Blends (NOFBX) propellants for manned and unmanned spaceflight applications. In L Ouwehand, editor, *Proceedings of the 5th IAASS Conference A Safer Space for Safer World*, number 67, January 2012.
[29] Greg Mungas, David Fisher, Joanne Vozoff, and Marco Villa. NOFBX single stage to orbit Mars ascent vehicle. In *Proceedings of the 2012 IEEE Aerospace Conference*, pages 1–11, March 3–10 2012.
[30] J M Vozoff, D J Fisher, and G S Mungas. NOFBX Mars ascent vehicle: A single stage to orbit approach. In *Proceedings of the Workshop on Concepts and Approaches for Mars Exploration*, number 4353, June 12–14 2012.
[31] Max Vozoff and Greg Mungas. NOFBX: A non-toxic, "green" propulsion technology with high performance and low cost. In *Proceedings of the AIAA SPACE 2012 Conference & Exposition*, number AIAA 2012-5235, September 11–13 2012.
[32] Gregory Mungas, David J Fisher, Christopher Mungas, and Benjamin Carryer. Nitrous oxide fuel blend monopropellants, May 28 2009. Patent application number: 20090133788.
[33] Benjamin B Donahue. Mars ascent-stage design utilizing nuclear propulsion. *Journal of Spacecraft and Rockets*, 32(3):552–558, May–June 1995.
[34] Satshi Kaneco, Kenji Iiba, Nobu-Hide Hiei, Kiyohisa Ohta, Takayuki Mizuno, and Tohru Suzuki. Electrochemical reduction of carbon dioxide to ethylene with high Faradaic efficiency at a Cu electrode in CsOH/methanol. *Electrochimica Acta*, 44(26):4701–4706, September 15 1999.
[35] Kai-Uwe Hinrichs, John M Hayes, Wolfgang Bach, Arthur J Spivack, Laura R Hmelo, Nils G Holm, Carl G Johnson, and Sean P Sylva. Biological formation of ethane and propane in the deep marine subsurface. *Proceedings of the National Academy of Sciences*, 103(40):14684–14689, October 3 2006.
[36] Raymond J Zeng, Zhiguo Yuan, and Jürg Keller. Enrichment of denitrifying glycogen-accumulating organisms in anaerobic/anoxic activated sludge system. *Biotechnology and Bioengineering*, 81(4):397–404, February 20 2003.
[37] Marlies J Kampschreur, Hardy Temmink, Robert Kleerebezem, Mike S M Jetten, and Mark C M van Loosdrecht. Nitrous oxide emission during wastewater treatment. *Water Research*, 43(17):4093–4103, September 2009.
[38] Lisa Y Stein. Surveying N_2O-producing pathways in bacteria. In M G Klotz, editor, *Methods in Enzymology: Nitrification and Related Processes*, volume 486, pages 131–152. Academic Press, Oxford, United Kingdom, 2011.
[39] Takashi Osada, Kazutaka Kuroda, and Michihiro Yonaga. Reducing nitrous oxide gas emissions from fill-and-draw type activated sludge process. *Water Research*, 29(6):1607–1608, June 1995.
[40] M Maag and F P Vinther. Nitrous oxide emission by nitrification and denitrification in different soil types and at different soil moisture contents and temperatures. *Applied Soil Ecology*, 4(1):5–14, July 1996.
[41] N Wrage, G L Velthof, M L van Beusichem, and O Oenema. Role of nitrifier denitrification in the production of nitrous oxide. *Soil Biology and Biochemistry*, 37(12-13):1723–1732, October 2001.
[42] Jan Willem van Groenigen, Peter J Kuikman, Willy J M de Groot, and Gerard L Velthof. Nitrous oxide emission from urine-treated soil as influenced by urine composition and soil physical conditions. *Soil Biology and Biochemistry*, 37(3):463–473, March 2005.
[43] Romain Lemaire, Rikke Meyer, Annelies Taske, Gregory R Crocetti, Jürg Keller, and Zhiguo Yuan. Identifying causes for N_2O accumulation in a lab-scale sequencing batch reactor performing simultaneous nitrification, denitrification and phosphorus removal. *Journal of Biotechnology*, 122(1):62–72, March 9 2006.
[44] Nouceiba Adouani, Thomas Lendormi, Lionel Limousy, and Olivier Sire. Effect of the carbon source on N_2O emissions during biological denitrification. *Resources, Conservation and Recycling*, 54(5):299–302, March 2010.
[45] Brian Anderson, Karen Bartlett, Steven Frolking, Katharine Hayhoe, Jennifer Jenkins, and William Salas. Methane and nitrous oxide emissions from natural sources. Technical Report EPA 430-R-10-001, United States Environmental Protection Agency, April 2010.
[46] Huijie Lu and Kartik Chandran. Factors promoting emissions of nitrous oxide and nitric oxide from denitrifying sequencing batch reactors operated with methanol and ethanol as electron donors. *Biotechnology and Bioengineering*, 106(3):390–398, June 15 2010.
[47] Wenlin Jia, Jian Zhang, Huijun Xie, Yujie Yan, Jinhe Wang, Yongxin Zhao, and Xiaoli Xu. Effect of PHB and oxygen uptake rate on nitrous oxide emission during simultaneous nitrification denitrification process. *Bioresource Technology*, 113:232–238, June 2012.
[48] Daniel Strain and Mark Shwartz. Stanford engineers use rocket science to make wastewater treatment sustainable. *Stanford Report*, July 26 2010. URL `http://news.stanford.edu/news/2010/july/waste-072610.html`.
